# An efficient 3-acylquinoline synthesis from acetophenones and anthranil *via* C(sp^3^)–H bond activation mediated by Selectfluor†

**DOI:** 10.1039/c9ra01481k

**Published:** 2019-04-02

**Authors:** Yejun Gao, Robert C. Hider, Yongmin Ma

**Affiliations:** School of Pharmaceutical and Chemical Engineering, Taizhou University Taizhou 318000 PR China yongmin.ma@tzc.edu.cn; School of Pharmaceutical Science, Zhejiang Chinese Medical University Hangzhou 310053 PR China; Institute of Pharmaceutical Science, King's College London Franklin-Wilkins Building, Stamford Street London SE1 9NH UK

## Abstract

An efficient method for the synthesis of 3-functionalized quinolines from commercially available acetophenones and anthranil has been described. Selectfluor propels the C(sp^3^)–H bond activation of the acetophenones and aza-Michael addition of anthranil resulting in annulated 3-acylquinolines in moderate to high yields. DMSO acts not only as a solvent but also as a one carbon donor in the reaction.

## Abstract 2

An efficient method for the synthesis of 3-functionalized quinolines from acetophenones and anthranil mediated by Selectfluor was achieved.

## Graphical abstract

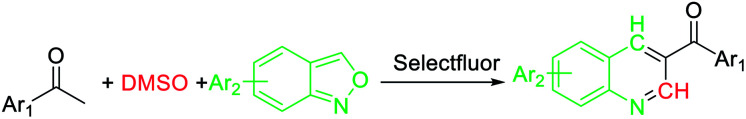

## Introduction

**Fig. 1:**
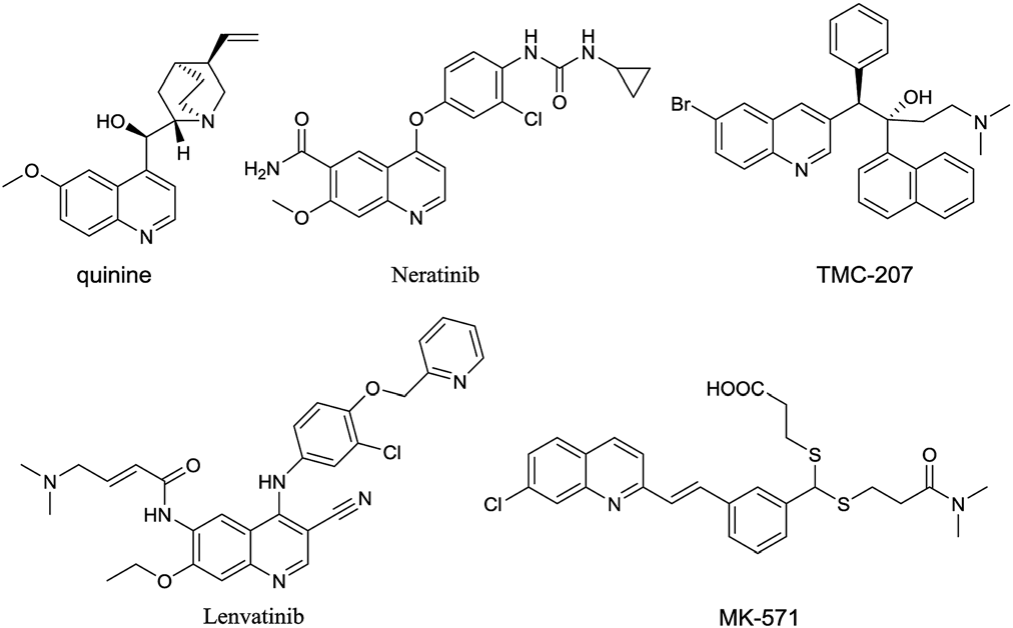
Examples of biological active quinoline derivatives.

Quinolines are one of the most ubiquitous structural units in natural products,^1–5^ biologically active compounds^6–11^ and functionalized materials (Fig. 1).^12–14^ The synthesis of quinolines has been an active area for many years and as a result, a number of efficient synthetic methods have been developed,^15–20^ such as the classical Skraup,^21–23^ Combes,^24–26^ Friedlander,^27–29^ Gould–Jacobs,^30–32^ and Doebner–von Miller reactions.^33–36^ More recently, alternative strategies for the quinoline synthesis such as domino cycloadditions^37–41^ and transition-metal mediated methods^42–45^ have been introduced. However, most of the existing methods necessitate the use of strong acid or base conditions, high reaction temperatures, the use of expensive/toxic metal catalysts and frequently require the use of expensive, highly functionalized starting materials. Such issues present serious problems from the environmental point of view and for industrial scale production. Therefore, the development of new synthetic protocols for the quinoline synthesis in sustainable, environmentally friendly and efficient fashion are highly desirable.

3-Acylquinolines have been reported to possess herbicidal activity^46^ as well as antihypertensive activity.^47^ Some efficient methods for the preparation of 3-acylquinolines have been developed, such as Pd-catalyzed carbonylative Suzuki cross-coupling reactions of arylboronic acid with 3-iodoquinoline,^48^ Pd-catalyzed coupling of aldehydes and 3-bromoquinoline^49^ or arylboronic acid and 3-quinolinecarbaldehyde,^50^ Fe-catalyzed cascade Michael addition/cyclization of *o*-aminoaryl aldehydes/ketones/alcohols with ynones,^45^ and domino reactions between *N*,*N*-dimethyl enaminones and anilines.^41^ In addition, dehydrogenation of saturated carbonyl compounds to afford α,β-unsaturated carbonyl derivatives has been found to be compatible with other organic transformations, leading to efficient one-pot protocols for the synthesis of functionalized molecules.^51–54^ Stimulated by this excellent pioneering work, Fan *et al.* developed a copper-catalyzed α,β functionalization of saturated ketones with 2-aminoaryl carbonyl compounds *via* a C(sp^3^)–H bond amination, enaminone formation, and enamine-carbonyl condensation process.^55^ Tiwari *et al.* reported a similar reaction starting from saturated ketones and anthranils *via* sequential dehydrogenation/aza-Michael addition/annulations cascade reactions in a one pot.^56^ The same group also reported the reaction anthranil is an isoxazole derivative which has been used for synthesis of a couple of heterocycles by cleavage of N–O bond.^57–61^ Inspired by these excellent pioneering work, especially Tiwari's report that quinolines can be transformed from the reaction of *in situ* generated α,β-unsaturated ketones from acetophenones *via* one-carbon homologation by DMSO followed by the aza-Michael addition of anthranils and subsequent annulation,^62^ we were encouraged to use Selectfluor^63–68^ as an alternative oxidant for the synthetic approach to 3-acylquinolines from commercially available acetophenones and anthranil. In this case, DMSO was applied as both carbon source and reaction medium. Herein, we wish to report the new protocol involving N–O bond cleavage and the formation of three C–C bonds.

## Results and discussion

To initiate the study, a reaction was carried out using acetophenone (1a) and anthranil (2a) as model substrates, the details being summarized in Table 1. We first performed the reaction with Selectfluor as an oxidant in DMSO, and a good yield (71%) was achieved when the reaction was carried out at 100 °C for 24 h (entry 1). In an attempt to improve the efficiency, the effect of various carbon donors such as DMF, *N*,*N*-dimethylacetamide (DMAC) and *N*-methylformamide (NMF) was investigated, but appreciably lower yields were obtained (entries 2–4). The subsequent brief investigation in varying the carbon source indicated that DMSO was a relatively ideal carbon source. Furthermore, the reaction did not proceed at all in toluene, a non-carbon donor solvent (entry 5). We then investigated the effect of oxidants on the transformation. (NH_4_)_2_S_2_O_8_ or K_2_S_2_O_8_ has been reported to efficiently oxidize acetophenones and other substrates,^69–72^ and Tiwari and co-workers declared that 37% and 76% yield of 3aa were obtained at 120 °C mediated by (NH_4_)_2_S_2_O_8_ and K_2_S_2_O_8_, respectively.^62^ However, we found that both (NH_4_)_2_S_2_O_8_ and K_2_S_2_O_8_ are much less efficient in our conditions as compared with Selectfluor (entries 6 and 7 *vs.* 1). In the absence of any oxidant, the desired product was not observed (entry 8). Increasing the reaction time to 48 h did not markedly improve the efficiency (entry 9). However, a significantly lower yield was achieved when the reaction was performed for 16 h or less, with a recovery of a large amount of the starting material (entries 10–12). Increasing or decreasing the reaction temperature away from 100 °C did not improve the product yield (entries 13–16 *vs.* 1). Based on these results, the optimal reaction conditions selected were acetophenone (1 mmol), anthranil (1 mmol) and Selectfluor (1.1 mmol) in DMSO (3 mL) at 100 °C in open air for 24 h.

**Table 1:** Optimization of the reaction conditions^a^

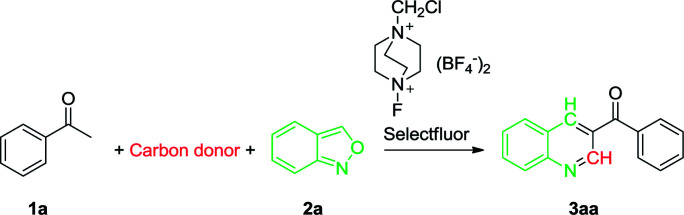					
Entry	Solvent	Oxidant	Time (h)	Temp (°C)	Yield (%)
1	DMSO	SF	24	100	71, 15^b^, 65^c^
2	DMF	SF	24	100	23
3	DMAC	SF	24	100	Trace
4	Toluene	SF	24	100	0
5	NMF	SF	24	100	18
6	DMSO	(NH_4)2_S_2_O_8_	24	100	Trace
7	DMSO	K_2_S_2_O_8_	24	100	33
8	DMSO	—	24	100	0
9	DMSO	SF	48	100	73
10	DMSO	SF	16	100	46
11	DMSO	SF	8	100	23
12	DMSO	SF	4	100	Trace
13	DMSO	SF	24	120	67
14	DMSO	SF	24	80	56
15	DMSO	SF	24	60	29
16	DMSO	SF	24	40	0

a Reaction conditions: 1a (1 mmol), 2 (1 mmol), oxidant (1.1 mmol) and DMSO (3 mL) in a sealed tube at the indicated reaction conditions.

b Reaction under N_2_.

c Reaction was performed on a 10 mmol scale of 1a.

With the optimal reaction conditions in hand, we progressed to explore the substrate scope of the aryl methyl ketones and anthranils in order to investigate the reaction generality. The results are summarized in Table 2. We first examined the substituent effect of aryl ketones with anthranil 2a. A variety of aryl methyl ketones were well tolerated with both electron-donating groups (–OMe, –Me, –*n*-Pr) and electron-withdrawing groups (–Br, –Cl, –F) on the aromatic ring, leading to the desired products in 48–74% yield (3aa–3da, 3ga–3ka, 3ma–3ra). The electronic nature of the aryl moiety had no marked effect on the product yield. However, their steric property did influence the yield appreciably. For instance, the average yield of the *ortho*-substituted 3aa–3da is 57% whereas the average yields of the *meta*- and *para*-substituted analogues (3ga–3ka and 3ma–3ra) are 63% and 64%, respectively. In addition, when a hydroxyl or amino group was introduced on the aromatic ring, the corresponding product failed to form (3ea, 3fa, 3la and 3sa). This may be due to the influence of an active proton on the reaction. However, when the amino group was protected with two methyl groups (–NMe_2_), the corresponding 3-acylquinoline (3ta) also failed to be formed. The desired product was also not prepared when a nitro substituted acetophenone was employed as the substrate (3ua).

**Table 2:** Substrate scope^a^^,^^b^

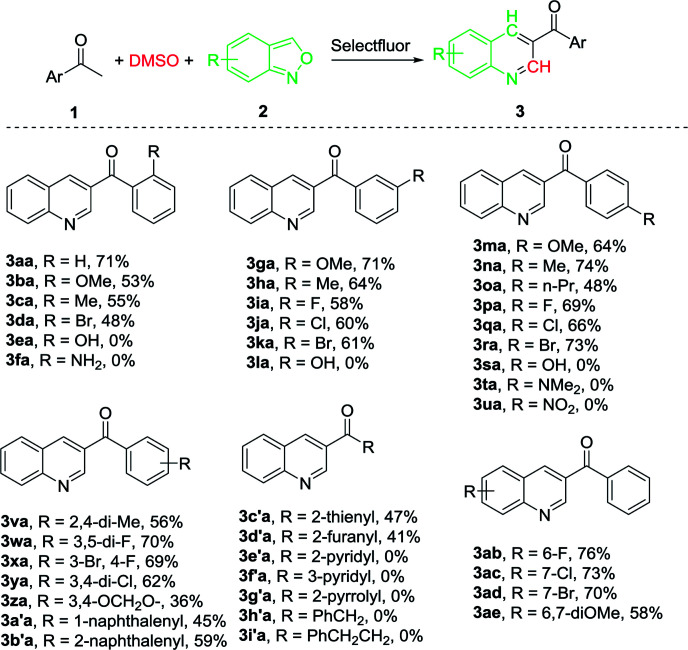


a Reaction conditions: 1 (1 mmol), 2 (1 mmol), Selectfluor (1.1 mmol), DMSO (3 mL), 100 °C, 24 h.

b Isolated yield.

We also investigated the reaction generality of di-substituted acetophenones and heterocyclic ketones. In similar fashion to mono-substituted analogues, the di-substituted methyl ketones reacted smoothly to furnish the corresponding 3-acylquinolines in an average yield of 57% (3va–3b′a) (Table 2). Again, steric hindrance influences the reaction yield dramatically. As a result, 59% and 45% yield were achieved when 1-(naphthalene-2-yl)ethanone and 1-(naphthalene-1-yl)ethanone were employed respectively (3a′a and 3b′a). With the heterocyclic ketones employed, only 2-thienyl ketone (1c′a) and 2-furanyl ketone (1d′) were successfully converted into the corresponding 3-heterocyclic quinolines in moderate yields (47% and 41% respectively) and the other three (1e′–1g′) failed. Aliphatic ketones such as 1-phenylpropan-2-one (1h′) and 4-phenylbutan-2-one (1i′) failed to provide the desired products.

The scope of substituted anthranils in this novel reaction was also investigated (Table 2). Anthranils bearing either an electron-withdrawing group (EWG) such as a fluoro, chloro and bromo group, or an electron-donating group (EDG) such as dimethoxy group on the aromatic ring, underwent this reaction smoothly to provide the corresponding quinolines in moderate to good yields (3ab-3ae). In this case, the introduction of the EWG did not influence the product yield (3ab–3ad*vs.*3aa) while the introduction of the EDG decreased the yield from 71% to 58% (3ae*vs.*3aa).

**Scheme 1:**
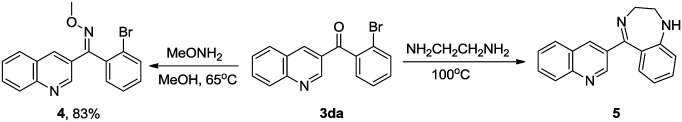
Conversion of 3-acylquinoline into biologically active molecules.

This initial success led us to further investigate the synthesis of biologically active molecules (Scheme 1). In this regard, both the ketone and halide groups are well tolerated and the method offers the chance for further diversification and amplification of the quinoline moiety. For example, the (2-bromophenyl)(quinolin-3-yl)methanone (3da) was heated in MeOH in the presence of methoxyamine to afford quinolinylmethanone oxime (4) as bactericidal agent for agricultural application.^73^3da was also reported to successively synthesize 2,3-dihydro-5-(3-quinolinyl)-1*H*-1,4-benzodiazepine (5) as a fungicide.^56,62,74^

**Scheme 2:**
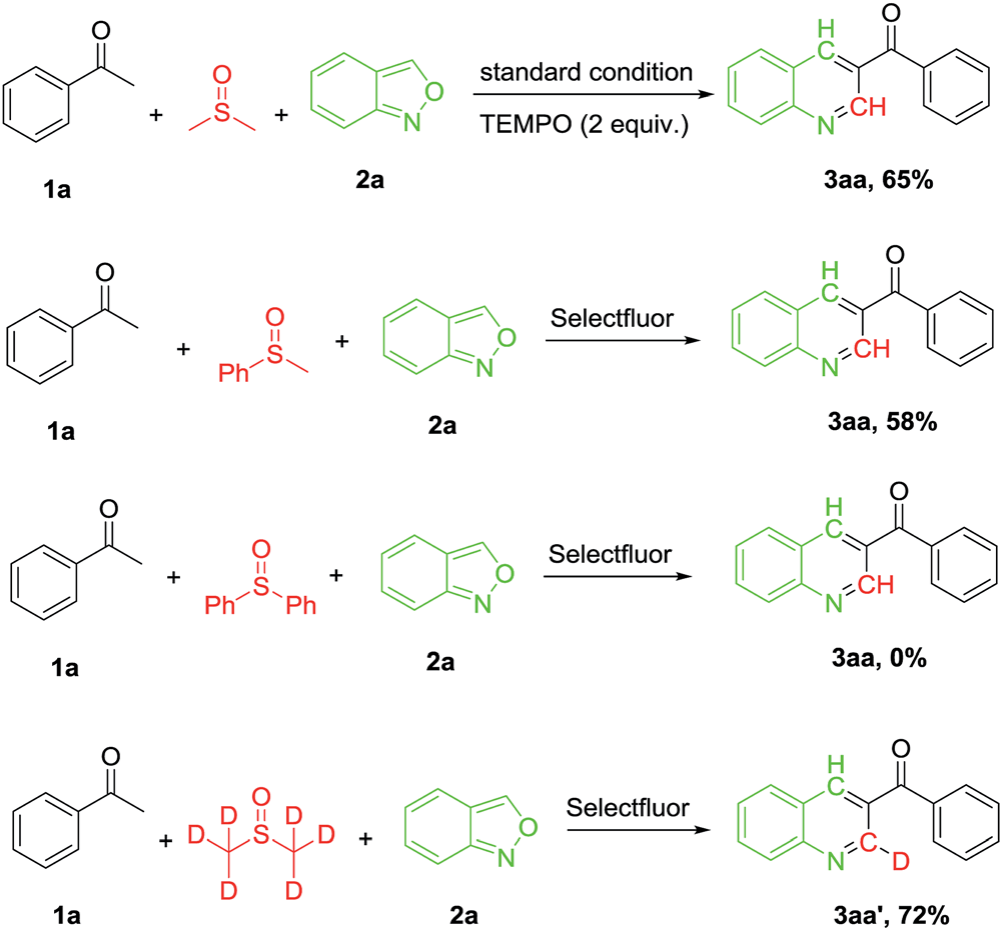
Control experiments.

Several control experiments were performed to gain a preliminary insight into the reaction mechanism (Scheme 2). When the reaction was carried out in the presence of TEMPO (2 equiv.), a similar yield (65%) of the desired product (3aa) was afforded (71% in the absence of TEMPO), indicating that a radical process may not be involved in the reaction. When the reaction was carried out under N_2_ atmosphere, the desired product was obtained at 15% yield only (Table 1, entry 1), suggesting that O_2_ was involved in the oxidative process. On the other hand, no markedly improved yield was given when the reaction was carried out under oxygen atmosphere. To verify the carbon source in the reaction from DMSO, the reaction was performed in methyl phenyl sulfoxide and diphenyl sulfoxide, the expected product 3aa was obtained in 58% and 0% yield, respectively. Furthermore, the reaction of 1a was performed with deuterium-labelling DMSO and a C2 deuterated 3aa′ was the sole product. This result confirmed that the carbon on the 2-position of the pyridine ring originated from DMSO.

To illustrate the suitability of this new synthetic method on an enlarged scale, a gram-scale experiment (10 mmol) was carried out. The reaction of 1a proceeded smoothly under standard conditions to afford the corresponding product 3aa in 65% yield (Table 1, entry 1) without an appreciable loss of efficiency (1 mmol, 71%).

**Scheme 3:**
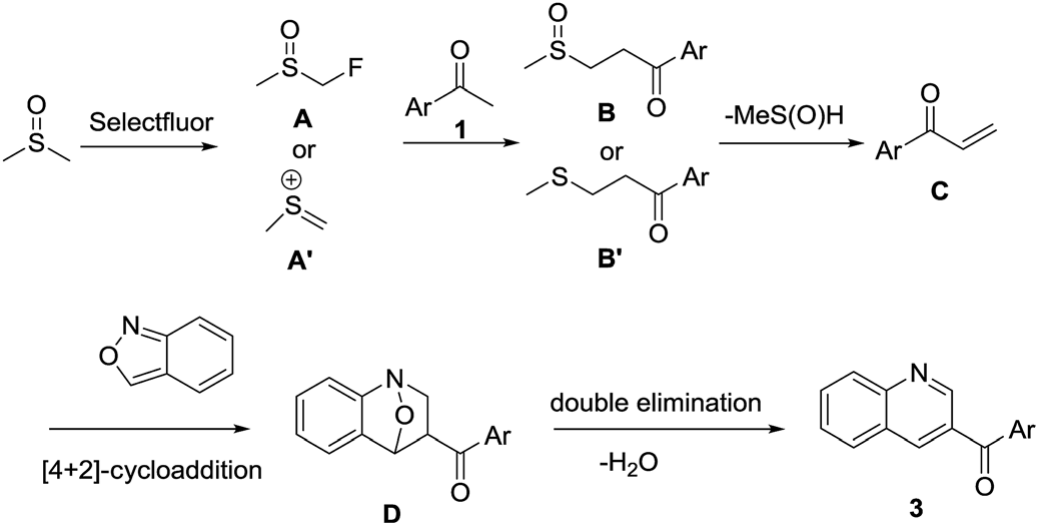
Proposed reaction mechanism.

On the basis of these preliminary experimental results and previous literature reports,^62,68^ a possible mechanism is proposed (Scheme 3). DMSO is initially activated by Selectfluor to furnish α-fluorinated intermediate A or methyl(methylene)sulfonium cation A′, which on reaction with acetophenone 1 furnishes intermediate B or B′, respectively. The intermediate B or B′ undergoes spontaneous demethylsulfinylation or demethylthioation to remove MeS(=O)H or MeSH, respectively. The intermediate C undergoes [4 + 2] cycloaddition with anthranil 2 to afford intermediate D which followed by double elimination to remove water to give the final product 3.

## Conclusions

In conclusion, we have successfully developed a Selectfluor-promoted efficient three-component cascade reaction procedure for the synthesis of 3-acylquinolines from readily available acetophenones, anthranils and DMSO. The reaction is compatible with various ketones and anthranils and DMSO is served as both solvent and a carbon source. Considering the wide availability of the substrates, broad substrate generality and the mild reaction conditions, the present work provides an attractive novel protocol for the synthesis of the 3-acylquinolines.

## Conflicts of interest

There are no conflicts to declare.

## Supplementary Material

RA-009-C9RA01481K-s001
