# Supplementary material for: An efficient 3-acylquinoline synthesis from acetophenones and anthranil via C(sp3)–H bond activation mediated by Selectfluor
Source: RSC Adv. 2019 Apr 2;9(18):10340–4. doi: 10.1039/c9ra01481k (PMC9062302; doi:10.1039/c9ra01481k)
Supplement: RA-009-C9RA01481K-s001 [file RA-009-C9RA01481K-s001.pdf]

Electronic Supplementary Material (ESI) for RSC Advances.
This journal is © The Royal Society of Chemistry 2019

An Efficient 3-Acylquinoline Synthesis from Acetophenones and Anthranil via C(sp^3)-H Bond Activation Mediated by Selectfluor

Yejun Gao[a,b], Robert C Hider[c] and Yongmin Ma[a,b*]

[a]School of Pharmaceutical and Chemical Engineering, Taizhou University, Taizhou 318000, P R China

[b]School of Pharmaceutical Science, Zhejiang Chinese Medical University, Hangzhou, 310053, P R China

[c]Institute of Pharmaceutical Science, King's College London, Franklin-Wilkins Building, Stamford Street, London, SE1 9NH, UK

*Corresponding author. Email: yongmin.ma@tzc.edu.cn

SUPPORTING INFORMATION

Experimental Section

All reagents and solvents were used from commercial sources unless otherwise stated. All raw materials are obtained from commercial sources or synthesized and used by themselves. All experiments were conducted in the air. Thin-layer chromatography (TLC) uses a pre-coated plate (silica gel 60 PF254, 0.25 mm or 0.5 mm). Column chromatography on silica gel (240-400 mesh) with petroleum ether and ethyl acetate as eluent. The 1H and 13C NMR spectra of the 200/400/600 MHz and 50/100/125 MHz NMR spectrometers were recorded in CDCl3/DMSO-d6, respectively. Chemical changes are reported as δ peaks. Uncorrected melting point. Mass spectra were obtained using an LC-MS (ESI) mass or GC-MS mass spectrometer.

Procedures

Selectfluor (390mg, 1.1 mmol), acetophenone (120 mg, 1 mmol) and anthranil (120 mg, 1 mmol) were added to a solution of DMSO (3.0 mL), the reaction mixture was stirred in a tube at 100 ^{o}C for 24 h. The reaction was monitored by TLC. Once the reaction was completed, the reaction mixture was treated with H_2O (15.0 mL) and EtOAc (8.0 mL). The organic and aqueous layers were then separated, and the aqueous layer was extracted with EtOAc (3 x 8 mL). The combined organic extracts were dried (Na_2SO_4), then the solvent was removed under reduced pressure and the remaining residue was purified by column chromatography. Compound **3aa** (166 mg, 71% yield) was obtained as a white solid. (3a as an Example)

Phenyl(quinolin-3-yl)methanone (3aa)

White solid; mp 73-75 °C (lit. value[1] 74-75 °C). ^{1}H NMR (500 MHz, $CDCl_3$) δ 9.33 (d, *J* = 2.2 Hz, 1H), 8.58 (d, *J* = 2.1 Hz, 1H), 8.22 (d, *J* = 8.5 Hz, 1H), 7.94 (dd, *J* = 8.1, 1.4 Hz, 1H), 7.86 – 7.88 (m, 3H), 7.69 – 7.64 (m, 2H), 7.57 – 7.53 (m, 2H). ^{13}C NMR (126 MHz, $CDCl_3$) δ 194.84, 150.27, 149.36, 138.93, 137.03, 133.11, 131.93, 130.11, 130.05, 129.43, 129.18, 128.68, 127.66, 126.66. EI-MS: m/z $[M+H]^+$ 234.

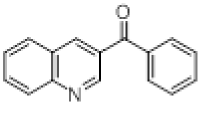

3aa

(2-Methoxyphenyl)(quinolin-3-yl)methanone (3ba)

Yellow solid; mp 73-75 °C. ^{1}H NMR (400 MHz, $CDCl_3$) δ 9.30 (d, *J* = 2.1 Hz, 1H), 8.63 – 8.59 (m, 1H), 8.24 – 8.19 (m, 1H), 7.99 – 7.94 (m, 1H), 7.89 – 7.86 (m, 1H), 7.68 – 7.55 (m, 3H), 7.17 (td, *J* = 7.5, 0.9 Hz, 1H), 7.08 (d, *J* = 8.4 Hz, 1H), 3.75 (s, 3H). ^{13}C NMR (101 MHz, $CDCl_3$) δ 194.77, 157.59, 150.47, 149.48, 138.57, 133.04, 131.82, 130.62, 130.25, 129.43, 129.33, 127.84, 127.34, 127.03, 121.01, 111.55, 55.58. HRMS (ESI): calcd. for $C_{17}H_{14}NO_2$ $[M + H]^+$ 264.1019; found 264.1008.

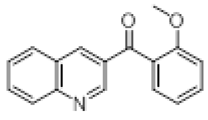

3ba

Quinolin-3-yl(o-tolyl)methanone (3ca)

Light yellow oil. ^{1}H NMR (500 MHz, $CDCl_3$) δ 9.36 (d, *J* = 2.2 Hz, 1H), 8.51 (d, *J* = 2.1 Hz, 1H), 8.22 (d, *J* = 8.5 Hz, 1H), 7.92 – 7.85 (m, 2H), 7.62 – 7.66 (m, 1H), 7.48 (td, *J* = 7.5, 1.5 Hz, 1H), 7.41 – 7.36 (m, 2H), 7.33 – 7.29 (m, 1H), 2.41 (s, 3H). ^{13}C NMR (126 MHz, $CDCl_3$) δ 196.85, 150.15, 149.45, 139.59, 137.50, 137.35, 132.27, 131.49, 131.04, 130.23, 129.40, 129.32, 128.91, 127.69, 126.78, 125.52, 20.17. HRMS (ESI): calcd. for $C_{17}H_{14}NO$ $[M + H]^+$ 248.1070; found 248.1077.

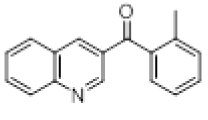

3ca

(2-Bromophenyl)(quinolin-3-yl)methanone (3da)

White solid; mp 131-133 °C (lit. value[2] 130-132 °C).^{1}H NMR (400 MHz, $CDCl_3$) δ 9.39 (d, *J* = 2.1 Hz, 1H), 8.54 (d, *J* = 2.2 Hz, 1H), 8.24 (d, *J* = 8.5 Hz, 1H), 7.97 – 7.89 (m, 2H), 7.78 – 7.74 (m, 1H), 7.68 (ddd, *J* = 8.1, 6.9, 1.1 Hz, 1H), 7.53 (dd, *J* = 6.7, 1.1 Hz, 1H), 7.49 (dt, *J* = 7.4, 1.4 Hz, 2H). ^{13}C NMR (101 MHz, $CDCl_3$) δ 194.53, 149.98, 149.75, 139.80, 139.74, 133.52, 132.55, 131.90, 129.56, 129.44, 129.33, 128.68, 127.75, 127.63, 126.83, 119.68. EI-MS: m/z $[M+H]^+$ 312.

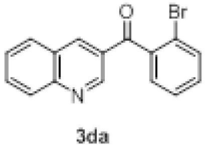

3da

(3-Methoxyphenyl)(quinolin-3-yl)methanone (3ga)

Yellow oil[2]. ^{1}H NMR (400 MHz, $CDCl_3$) δ 9.38 (d, *J* = 2.1 Hz, 1H), 8.64 (d, *J* = 2.2 Hz, 1H), 8.27 (d, *J* = 8.5 Hz, 1H), 8.02 – 7.97 (m, 1H), 7.94 – 7.89 (m, 1H), 7.73 – 7.69 (m, 1H), 7.50 – 7.44 (m, 3H), 7.27 – 7.23 (m, 1H), 3.93 (s, 3H). ^{13}C NMR (101 MHz, $CDCl_3$) δ 194.58, 159.91, 150.13, 149.17, 139.07,

138.30, 132.04, 130.20, 129.63, 129.30, 129.21, 127.73, 126.68, 122.85, 119.62, 114.24, 55.56. EI-MS: m/z $[M+H]^+$ 264.

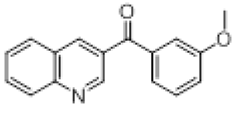

3ga

Quinolin-3-yl(m-tolyl)methanone (3ha)

Light yellow oil. ^{1}H NMR (400 MHz, $CDCl_3$) δ 9.37 (d, *J* = 2.1 Hz, 1H), 8.64 (d, *J* = 2.1 Hz, 1H), 8.27 (d, *J* = 8.5 Hz, 1H), 7.99 (dd, *J* = 8.2, 1.4 Hz, 1H), 7.94 – 7.91 (m, 1H), 7.75 – 7.68 (m, 3H), 7.52 – 7.48 (m, 2H), 2.50 (s, 3H). ^{13}C NMR (101 MHz, $CDCl_3$) δ 195.01, 150.23, 149.21, 138.98, 138.68, 137.07, 133.92, 131.95, 130.46, 130.34, 129.34, 129.20, 128.50, 127.67, 127.37, 126.74, 21.40. HRMS (ESI): calcd. for $C_{17}H_{14}NO$ $[M + H]^+$ 248.1070; found 248.1067.

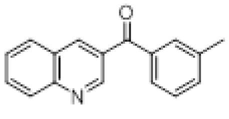

3ha

(3-Fluorophenyl)(quinolin-3-yl)methanone (3ia)

Light yellow oil. ^{1}H NMR (400 MHz, $CDCl_3$) δ 9.37 (d, *J* = 2.2 Hz, 1H), 8.62 (d, *J* = 2.2 Hz, 1H), 8.27 (d, *J* = 8.5 Hz, 1H), 7.99 (dd, *J* = 8.2, 1.4 Hz, 1H), 7.93 (ddd, *J* = 8.4, 6.9, 1.4 Hz, 1H), 7.74 – 7.66 (m, 2H), 7.65 – 7.55 (m, 2H), 7.43 – 7.39 (m, 1H). ^{13}C NMR (101 MHz, $CDCl_3$) δ 193.45, 163.22 (d, J_{C-F} = 248 Hz), 150.02, 149.45, 139.02, 132.21, 130.41 (d, J_{C-F} = 8 Hz), 129.60, 129.45, 129.24, 127.84, 126.59, 125.86, 120.18 (d, *J* = 21 Hz), 116.84 (d, *J* = 22 Hz). HRMS (ESI): calcd. for $C_{16}H_{11}FNO$ $[M + H]^+$ 252.0819; found 252.0839.

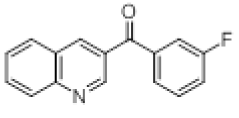

3ia

(3-Chlorophenyl)(quinolin-3-yl)methanone (3ja)

Light yellow oil. ^{1}H NMR (500 MHz, $CDCl_3$) δ 9.32 (d, *J* = 2.1 Hz, 1H), 8.57 (d, *J* = 2.2, 1H), 8.25 – 8.20 (m, 1H), 7.96 (dd, *J* = 8.2, 1.4 Hz, 1H), 7.91 – 7.85 (m, 2H), 7.75 – 7.71 (m, 1H), 7.68 – 7.65 (m, 1H), 7.64 – 7.62 (m, 1H), 7.52 – 7.48 (m, 1H). ^{13}C NMR (126 MHz, $CDCl_3$) δ 193.44, 149.98, 149.45, 139.05, 138.63, 135.07, 133.06, 132.25, 130.00, 129.86, 129.52, 129.44, 129.26, 128.09, 127.85, 126.59. HRMS (ESI): calcd. for $C_{16}H_{11}ClNO$ $[M + H]^+$ 268.0524; found 268.0512.

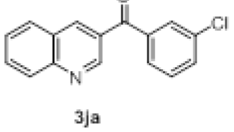

3ja

(3-Bromophenyl)(quinolin-3-yl)methanone (3ka)

Light yellow solid; mp 67-68 °C (lit. value[2] 67-69 °C). ^{1}H NMR (400 MHz, $CDCl_3$) δ 9.37 (d, *J* = 2.1 Hz, 1H), 8.63 (d, *J* = 2.1 Hz, 1H), 8.28 (d, *J* = 8.5 Hz, 1H), 8.06 (d, *J* = 1.8 Hz, 1H), 8.03 – 7.99 (m, 1H), 7.96 – 7.92 (m, 1H), 7.86 – 7.80 (m, 2H), 7.75 – 7.71 (m, 1H), 7.48 (t, *J* = 7.9 Hz, 1H). ^{13}C NMR (101 MHz, $CDCl_3$) δ 193.32, 149.94, 149.41, 139.13, 138.85, 136.00, 132.77, 132.30, 130.25, 129.52, 129.42, 129.29, 128.54, 127.90, 126.64, 123.06. EI-MS: m/z $[M+H]^+$ 312.

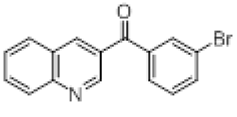

3ka

(4-Methoxyphenyl)(quinolin-3-yl)methanone (3ma)

White solid; mp 102-104 °C (lit. value[3] 103-105 °C). ^{1}H NMR (500 MHz, $CDCl_3$) δ 9.28 (d, *J* = 2.2 Hz, 1H), 8.54 (d, *J* = 2.2 Hz, 1H), 8.21 (dd, *J* = 8.5, 1.0 Hz, 1H), 7.93 (dd, *J* = 8.1, 1.3 Hz, 1H), 7.91 – 7.88 (m, 2H), 7.87 – 7.84 (m, 1H), 7.67 – 7.63 (m, 1H), 7.05 – 6.99 (m, 2H), 3.92 (s, 3H). ^{13}C NMR (126 MHz, $CDCl_3$) δ 193.43, 163.75, 150.23, 149.17, 138.33, 132.58, 131.64, 130.84, 129.70, 129.40, 129.04, 127.57, 126.73, 113.96, 55.61. EI-MS: m/z $[M+H]^+$ 264.

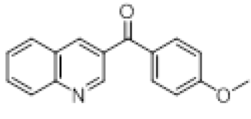

3ma

Quinolin-3-yl(p-tolyl)methanone (3na)

White solid; mp 88-89 °C (lit. value[3] 89-90 °C). ^{1}H NMR (500 MHz, $CDCl_3$) δ 9.31 (d, *J* = 2.1 Hz, 1H), 8.56 (d, *J* = 2.2 Hz, 1H), 8.21 (d, *J* = 8.4 Hz, 1H), 7.93 (dd, *J* = 8.2, 1.3 Hz, 1H), 7.88 – 7.84 (m, 1H), 7.82 – 7.77 (m, 2H), 7.67 – 7.63 (m, 1H), 7.35 (d, *J* = 7.9 Hz, 2H), 2.48 (s, 3H). ^{13}C NMR (126 MHz, $CDCl_3$) δ 194.50, 150.29, 149.26, 144.11, 138.70, 134.37, 131.78, 130.47, 130.29, 129.40, 129.37, 129.12, 127.60, 126.69, 21.75. EI-MS: m/z $[M+H]^+$ 248.

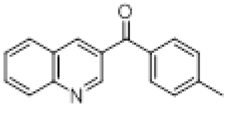

3na

(4-Propylphenyl)(quinolin-3-yl)methanone (3oa)

Yellow oil. ^{1}H NMR (400 MHz, DMSO-d_6) δ 9.22 (d, *J* = 2.2 Hz, 1H), 8.78 (d, *J* = 2.4 Hz, 1H), 8.23 (dd, *J* = 8.2, 1.4 Hz, 1H), 8.16 (dd, *J* = 8.4, 1.1 Hz, 1H), 7.98 – 7.94 (m, 1H), 7.87 – 7.82 (m, 2H), 7. 78 – 7.74 (m, 1H), 7.47 (d, *J* = 8.1 Hz, 2H), 2.72 (t, *J* = 7.6 Hz, 2H), 1.75 – 1.63 (m, 2H), 0.97 (t, *J* = 7.3 Hz, 3H). ^{13}C NMR (101 MHz, DMSO-d_6) δ 194.54, 150.24, 149.19, 148.70, 138.91, 134.73, 132.40, 130.60, 130.43, 130.23, 129.26, 128.06, 126.72, 37.69, 24.21, 14.14. HRMS (ESI): calcd. for $C_{19}H_{18}NO$ $[M + H]^+$ 276.1383; found 276.1371.

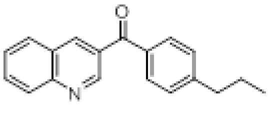

3oa

(4-Fluorophenyl)(quinolin-3-yl)methanone (3pa)

White solid; mp 80-81 °C (lit. value[2] 80-82 °C). ^{1}H NMR (400 MHz, $CDCl_3$) δ 9.34 (d, *J* = 2.2 Hz, 1H), 8.59 (d, *J* = 2.2 Hz, 1H), 8.26 (d, *J* = 8.5 Hz, 1H), 8.00 – 7.91 (m, 4H), 7.74 – 7.69 (m, 1H), 7.27 (d, *J* = 8.5 Hz, 2H). ^{13}C NMR (101 MHz, $CDCl_3$) δ 193.27, 165.79 (d, J_{C-F} = 254 Hz), 150.02, 149.36, 138.71, 133.35, 132.69 (d, J_{C-F} = 9 Hz), 132.03, 130.07, 129.44, 129.14, 127.78, 126.64, 115.94 (d, J_{C-F} = 22 Hz). EI-MS: m/z $[M+H]^+$ 252.

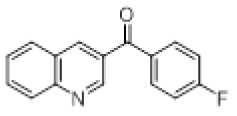

3pa

(4-Chlorophenyl)(quinolin-3-yl)methanone (3qa)

White solid; mp 105-106 °C (lit. value[2] 104-106 °C). ^{1}H NMR (500 MHz, $CDCl_3$) δ 9.30 (d, *J* = 2.3 Hz, 1H), 8.54 (d, *J* = 2.2 Hz, 1H), 8.21 (d, *J* = 8.5 Hz, 1H), 7.93 (dd, *J* = 8.2, 1.4 Hz, 1H), 7.90 – 7.86 (m, 1H), 7.84 – 7.81 (m, 2H), 7.68 – 7.64 (m, 1H), 7.58 – 7.50 (m, 2H). ^{13}C NMR (126 MHz, $CDCl_3$) δ 193.63, 150.07, 149.51, 139.68, 138.77, 135.33, 132.07, 131.42, 129.77, 129.52, 129.17, 129.05, 127.77, 126.58. EI-MS: m/z $[M+H]^+$ 268.

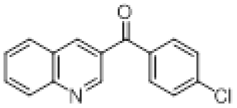

3qa

(4-Bromophenyl)(quinolin-3-yl)methanone (3ra)

White solid; mp 114-116 °C (lit. value[3] 115-117 °C). ^{1}H NMR (500 MHz, $CDCl_3$) δ 9.30 (d, *J* = 2.2 Hz, 1H), 8.56 (d, *J* = 2.2 Hz, 1H), 8.25 – 8.20 (m, 1H), 7.94 (dd, *J* = 8.2, 1.4 Hz, 1H), 7.91 – 7.87 (m, 1H), 7.75 (dt, *J* = 6.6, 2.0 Hz, 2H), 7.71 – 7.67 (m, 3H). ^{13}C NMR (126 MHz, $CDCl_3$) δ 193.68, 149.91, 149.26, 138.99, 135.70, 132.21, 132.05, 131.49, 129.72, 129.33, 129.19, 128.37, 127.87, 126.61. EI-MS: m/z $[M+H]^+$ 312.

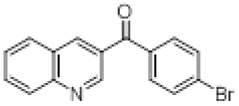

3ra

(2,4-Dimethylphenyl)(quinolin-3-yl)methanone (3va)

White solid; mp 118-120 °C. ^{1}H NMR (400 MHz, $CDCl_3$) δ 9.38 (d, *J* = 2.1 Hz, 1H), 8.54 (d, *J* = 2.1 Hz, 1H), 8.24 (d, *J* = 8.5 Hz, 1H), 7.95 – 7.87 (m, 2H), 7.69 – 7.65 (m, 1H), 7.35 (d, *J* = 7.8 Hz, 1H), 7.23 (s, 1H), 7.14 (d, *J* = 7.8 Hz, 1H), 2.46 (s, 3H), 2.45 (s, 3H). ^{13}C NMR (101 MHz, $CDCl_3$) δ 196.64, 150.30, 149.42, 141.64, 139.30, 137.93, 134.61, 132.40, 132.04, 130.76, 129.69, 129.34, 129.32, 127.59, 126.81, 126.11, 21.46, 20.27. HRMS (ESI): calcd. for $C_{18}H_{16}NO$ $[M + H]^+$ 262.1226; found 262.1238.

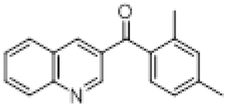

3va

(3,5-Difluorophenyl)(quinolin-3-yl)methanone (3wa)

White solid; mp 118-119 °C (lit. value[2] 118-120 °C). ^{1}H NMR (400 MHz, $CDCl_3$) δ 9.36 (d, J = 2.2 Hz, 1H), 8.61 (d, J = 2.2 Hz, 1H), 8.26 (dd, J = 8.5, 1.1 Hz, 1H), 8.00 (dd, J = 8.2, 1.4 Hz, 1H), 7.96 – 7.92 (m, 1H), 7.75 – 7.71 (m, 1H), 7.47 – 7.39 (m, 2H), 7.18 – 7.14 (m, 1H). ^{13}C NMR (101 MHz, $CDCl_3$) δ 192.18, 163.05 (d, J_{C-F} = 260 Hz), 149.81, 149.66, 138.98, 132.40, 129.56, 129.28, 128.97, 127.95, 126.49, 112.94 (d, J_{C-F} = 26 Hz), 112.94 (d, J_{C-F} = 11 Hz), 108.40 (t, J_{C-F} = 21 Hz). EI-MS: m/z $[M+H]^+$ 270.

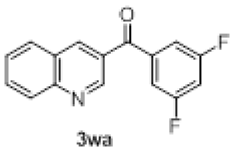

3wa

(3-Bromo-4-fluorophenyl)(quinolin-3-yl)methanone (3xa)

Yellow solid; mp 148-149 °C. ^{1}H NMR (400 MHz, $CDCl_3$) δ 9.34 (d, J = 1.8 Hz, 1H), 8.67 – 8.59 (m, 1H), 8.29 (t, J = 7.9 Hz, 1H), 8.17 (dt, J = 6.5, 2.0 Hz, 1H), 8.05 – 7.92 (m, 2H), 7.89 – 7.85 (m, 1H), 7.79 – 7.70 (m, 1H), 7.37 – 7.31 (m, 1H). ^{13}C NMR (101 MHz, $CDCl_3$) δ 192.07, 162.04 (d, J_{C-F} = 254 Hz), 149.81, 149.47, 138.82, 135.67, 134.51, 132.27, 131.19 (d, J_{C-F} = 8 Hz), 129.46, 129.21, 127.91, 126.57, 116.80 (d, J_{C-F} = 23 Hz), 110.01 (d, J_{C-F} = 21 Hz). HRMS (ESI): calcd. for $C_{16}H_{10}BrFNO$ $[M + H]^+$ 329.9924; found 329.9911.

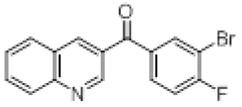

3xa

(3,4-Dichlorophenyl)(quinolin-3-yl)methanone (3ya)

Yellow solid; mp 115-116 °C (lit. value[1] 113-114 °C). ^{1}H NMR (400 MHz, $CDCl_3$) δ 9.34 (d, J = 2.1 Hz, 1H), 8.61 (d, J = 2.2 Hz, 1H), 8.28 (d, J = 8.5 Hz, 1H), 8.05 – 7.98 (m, 2H), 8.00 – 7.92 (m, 1H), 7.75 – 7.66 (m, 3H). ^{13}C NMR (101 MHz, $CDCl_3$) δ 192.37, 149.72, 149.44, 138.99, 137.86, 136.59, 133.55, 132.39, 131.76, 130.84, 129.43, 129.30, 129.24, 128.96, 127.97, 126.58. EI-MS: m/z $[M+H]^+$ 302.

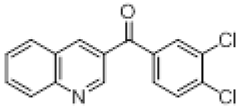

3ya

Benzo[d][1,3]dioxol-5-yl(quinolin-3-yl)methanone (3za)

White solid; mp 92-93 °C (lit. value[3] 92-94 °C). ^{1}H NMR (400 MHz, $CDCl_3$) δ 9.31 (d, J = 2.1 Hz, 1H), 8.62 – 8.53 (m, 1H), 8.25 (d, J = 8.5 Hz, 1H), 8.02 – 7.86 (m, 2H), 7.69 (t, J = 7.5 Hz, 1H), 7.46 (d, J = 6.2 Hz, 2H), 6.95 (d, J = 8.2 Hz, 1H), 6.15 (s, 2H). ^{13}C NMR (101 MHz, $CDCl_3$) δ 192.99, 152.17, 150.03, 149.04, 148.40, 138.48, 131.82, 131.46, 130.76, 129.30, 129.08, 127.70, 127.08, 109.71, 108.02, 102.10, 29.70. HRMS (ESI): calcd. for $C_{17}H_{12}NO_3$ $[M + H]^+$ 278.0812; found 278.0829.

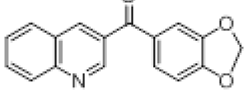

3za

Naphthalen-1-yl(quinolin-3-yl)methanone (3a'a)

pale-yellow oil[1]. ^{1}H NMR (400 MHz, $CDCl_3$) δ 9.49 – 9.44 (m, 1H), 8.68 – 8.66 (m, 1H), 8.33 (d, J = 8.3 Hz, 1H), 8.27 – 8.23 (m, 1H), 8.15 (d, J = 8.2 Hz, 1H), 8.05 – 8.01 (m, 1H), 7.95 (d, J = 7.6 Hz, 2H), 7.73 – 7.69 (m, 2H), 7.65 – 7.60 (m, 3H). ^{13}C NMR (101 MHz, $CDCl_3$) δ 195.96, 149.92, 148.95, 140.17, 135.17, 133.92, 132.56, 132.28, 130.95, 130.87, 129.45, 128.98, 128.62, 128.49, 127.90, 127.81, 126.83, 125.45. EI-MS: m/z $[M+H]^+$ 284.

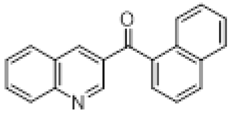

3a'a

Naphthalen-2-yl(quinolin-3-yl)methanone (3b'a)

Yellow solid[3]. ^{1}H NMR (400 MHz, $CDCl_3$) δ 9.43 (d, *J* = 2.2 Hz, 1H), 8.69 – 8.65 (m, 1H), 8.37 (d, *J* = 1.4 Hz, 1H), 8.31 – 8.26 (m, 1H), 8.05 (d, *J* = 1.3 Hz, 2H), 8.00 – 7.96 (m, 3H), 7.94 – 7.90 (m, 1H), 7.72 – 7.68 (m, 2H), 7.66 – 7.61 (m, 1H). ^{13}C NMR (101 MHz, $CDCl_3$) δ 194.81, 150.36, 149.46, 138.81, 135.54, 134.34, 132.31, 132.09, 131.86, 130.48, 129.51, 129.20, 128.77, 127.92, 127.64, 127.10, 126.73, 125.45. EI-MS: m/z $[M+H]^+$ 284.

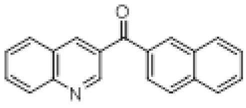

3b'a

Quinolin-3-yl(thiophen-2-yl)methanone (3c'a)

White solid; mp 88-90 °C (lit. value[3] 89-90 °C). ^{1}H NMR (400 MHz, $CDCl_3$) δ 9.40 (d, *J* = 2.2 Hz, 1H), 8.73 (d, *J* = 2.2 Hz, 1H), 8.26 (d, *J* = 8.5 Hz, 1H), 8.04 – 7.99 (m, 1H), 7.95 – 7.90 (m, 1H), 7.86 (dd, *J* = 5.0, 1.1 Hz, 1H), 7.78 (dd, *J* = 3.8, 1.1 Hz, 1H), 7.73 – 7.68 (m, 1H), 7.28 (dd, *J* = 4.9, 3.8 Hz, 1H). ^{13}C NMR (101 MHz, $CDCl_3$) δ 186.14, 149.58, 149.40, 146.13, 143.23, 137.85, 135.09, 131.85, 130.77, 129.46, 129.10, 128.36, 127.74, 126.74. EI-MS: m/z $[M+H]^+$ 240.

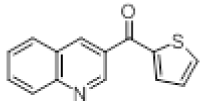

3c'a

Furan-2-yl(quinolin-3-yl)methanone (3d'a)

White solid; mp 100-101 °C (lit. value[3] 98-100 °C). ^{1}H NMR (500 MHz, $CDCl_3$) δ 9.47 (d, *J* = 2.3 Hz, 1H), 8.85 (d, *J* = 2.2 Hz, 1H), 8.22 – 8.17 (m, 1H), 7.98 (dd, *J* = 8.2, 1.4 Hz, 1H), 7.88 – 7.84 (m, 1H), 7.80 – 7.76 (m, 1H), 7.67 – 7.63 (m, 1H), 7.40 (dd, *J* = 3.6, 0.8 Hz, 1H), 6.68 (dd, *J* = 3.5, 1.7 Hz, 1H). ^{13}C NMR (126 MHz, $CDCl_3$) δ 180.36, 152.43, 149.78, 149.51, 147.48, 138.37, 131.92, 129.68, 129.46, 129.27, 127.59, 126.78, 120.77, 112.69. EI-MS: m/z $[M+H]^+$ 224.

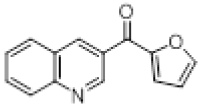

3d'a

(6-Fluoroquinolin-3-yl)(phenyl)methanone (3ab)

Yellow oil. ^{1}H NMR (400 MHz, $CDCl_3$) δ 9.31 (d, *J* = 2.1 Hz, 1H), 8.55 (d, *J* = 2.1 Hz, 1H), 8.25 (dd, *J* = 9.2, 5.2 Hz, 1H), 7.93 – 7.88 (m, 2H), 7.74 – 7.63 (m, 2H), 7.63 – 7.55 (m, 3H). ^{13}C NMR (101 MHz, $CDCl_3$) δ 194.62, 160.97 (d, $J_{C\text{-}F}$ = 249 Hz), 149.57, 146.46, 138.07, 136.82, 133.31, 131.99 (d, $J_{C\text{-}F}$ = 9 Hz), 130.80, 130.08, 128.75, 127.45 (d, $J_{C\text{-}F}$ = 10 Hz), 122.11 (d, $J_{C\text{-}F}$ = 26 Hz), 112.17 (d, $J_{C\text{-}F}$ = 22 Hz). HRMS (ESI): calcd. for $C_{16}H_{11}FNO$ $[M + H]^+$ 252.0819; found 252.0802.

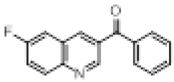

3ab

(7-Chloroquinolin-3-yl)(phenyl)methanone (3ac)

Yellow solid, mp 84-85 °C. ^{1}H NMR (400 MHz, CDCl$_3$) δ 9.37 (d, *J* = 2.1 Hz, 1H), 8.61 (d, *J* = 2.1 Hz, 1H), 8.27 (d, *J* = 2.0 Hz, 1H), 7.93 – 7.89 (m, 3H), 7.75 – 7.70 (m, 1H), 7.66 (dd, *J* = 8.7, 2.1 Hz, 1H), 7.60 (t, *J* = 7.7 Hz, 2H). ^{13}C NMR (101 MHz, CDCl$_3$) δ 194.30, 151.78, 151.10, 149.26, 138.91, 138.32, 136.77, 133.33, 130.34, 130.04, 129.03, 128.79, 128.31, 125.15. HRMS (ESI): calcd. for $C_{16}H_{11}ClNO$ [M + H]$^+$ 268.0524; found 268.0534.

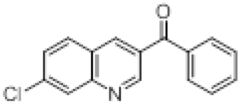

3ac

(7-Bromoquinolin-3-yl)(phenyl)methanone (3ad)

Yellow solid; mp 101-102 °C; ^{1}H NMR (400 MHz, CDCl$_3$) δ 9.36 (d, *J* = 2.1 Hz, 1H), 8.61 (d, *J* = 2.1 Hz, 1H), 8.47 (d, *J* = 1.9 Hz, 1H), 7.92 – 7.88 (m, 2H), 7.86 (d, *J* = 8.6 Hz, 1H), 7.80 (dd, *J* = 8.7, 1.8 Hz, 1H), 7.72 (td, *J* = 7.3, 1.4 Hz, 1H), 7.62 – 7.58 (m, 2H). ^{13}C NMR (101 MHz, CDCl$_3$) δ 194.24, 150.93, 139.07, 136.75, 133.34, 131.60, 130.45, 130.28, 130.03, 129.08, 129.00, 128.80, 126.80, 125.42. HRMS (ESI): calcd. for $C_{16}H_{11}BrNO$ [M + H]$^+$ 312.0019; found 312.0023.

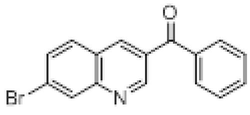

3ad

(6,7-Dimethoxyquinolin-3-yl)(phenyl)methanone (3ae)

White solid; mp 142-143 °C (lit. value[3] 143-145 °C). ^{1}H NMR (400 MHz, CDCl$_3$) δ 9.19 (d, *J* = 4.3 Hz, 1H), 8.50 (dt, *J* = 4.0, 2.1 Hz, 1H), 7.92 – 7.88 (m, 2H), 7.74 – 7.67 (m, 1H), 7.62 – 7.54 (m, 3H), 7.19 (dd, *J* = 4.3, 2.0 Hz, 1H), 4.14 (s, 3H), 4.08 (s, 3H). ^{13}C NMR (101 MHz, CDCl$_3$) δ 195.01, 154.62, 150.65, 148.38, 146.93, 137.44, 137.03, 132.79, 129.97, 128.58, 122.46, 107.80, 106.10, 56.40, 56.20. EI-MS: m/z [M+H]$^+$ 294.

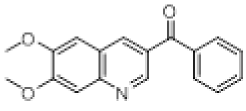

3ae

Phenyl(quinolin-3-yl-2-d)methanone (3aa')

White solid, mp 33-34 °C. ^{1}H NMR (400 MHz, CDCl$_3$) δ 8.60 (d, *J* = 2.4 Hz, 1H), 8.24 (d, *J* = 8.5 Hz, 1H), 7.96 (dd, *J* = 8.2, 1.4 Hz, 1H), 7.93 – 7.89 (m, 3H), 7.73 – 7.65 (m, 2H), 7.60 – 7.56 (m, 2H). ^{13}C NMR (101 MHz, CDCl$_3$) δ 194.87, 150.32, 149.42, 138.92, 137.06, 133.12, 131.92, 130.07, 129.47, 129.20, 128.69, 127.66, 126.68. HRMS (ESI): calcd. for $C_{16}H_{11}DNO$ [M + H]$^+$ 235.0976; found 235.0962.

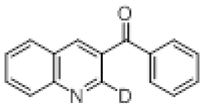

3aa'

(E)-(2-bromophenyl)(quinolin-3-yl)methanone O-methyl oxime (4)

White solid. ^{1}H NMR (400 MHz, CDCl$_3$) δ 9.39 (s, 1H), 8.17 (d, *J* = 8.5 Hz, 1H), 7.89 (s, 1H), 7.80 – 7.73 (m, 3H), 7.60 – 7.49 (m, 2H), 7.41 (td, *J* = 7.8, 1.8 Hz, 1H), 7.29 (dd, *J* = 7.6, 1.7 Hz, 1H), 4.11 (s, 3H). ^{13}C NMR (101 MHz, CDCl$_3$) δ 153.36, 148.54, 147.94, 134.56, 134.28, 133.60, 133.10, 131.98, 130.51, 130.19, 129.94, 129.29, 128.39, 127.63, 127.14, 121.83, 63.14. HRMS (ESI): calcd.

for $C_{17}H_{14}BrN_2O$ [M + H]$^+$ 241.0284; found 241.0294.

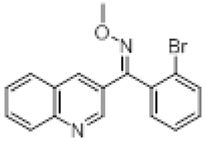

4

1. S. Khong and O. Kwon, *J. Org. Chem.*, 2012, **77**, 8257-8267.
2. S. B. Wakade, D. K. Tiwari, P. Ganesh, M. Phanindrudu, P. R. Likhar and D. K. Tiwari, *Org. Lett.*, 2017, **19**, 4948-4951.
3. D. K. Tiwari, M. Phanindrudu, S. B. Wakade, J. B. Nanubolu and D. K. Tiwari, *Chem. Commun.*, 2017, **53**, 5302-5305.

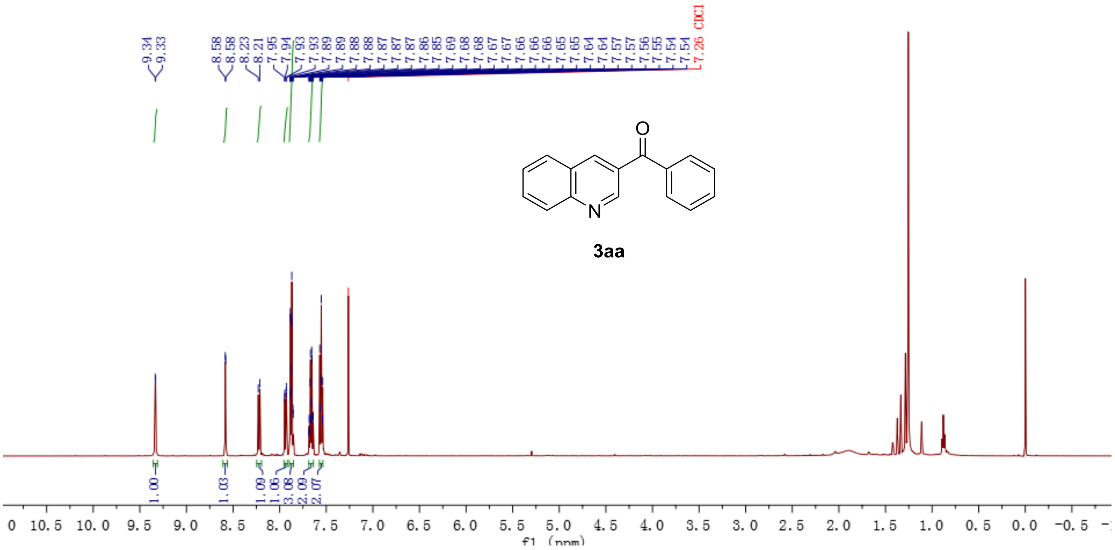
9.34
9.33
8.58
8.58
8.23
8.21
7.95
7.94
7.93
7.93
7.89
7.89
7.88
7.88
7.87
7.87
7.87
7.86
7.85
7.69
7.68
7.68
7.67
7.67
7.66
7.66
7.66
7.65
7.65
7.64
7.64
7.57
7.57
7.56
7.55
7.54
7.54
7.26 CDCl
O
N
3aa
1.00
1.03
1.09
1.06
3.08
2.09
2.07
f1 (ppm)

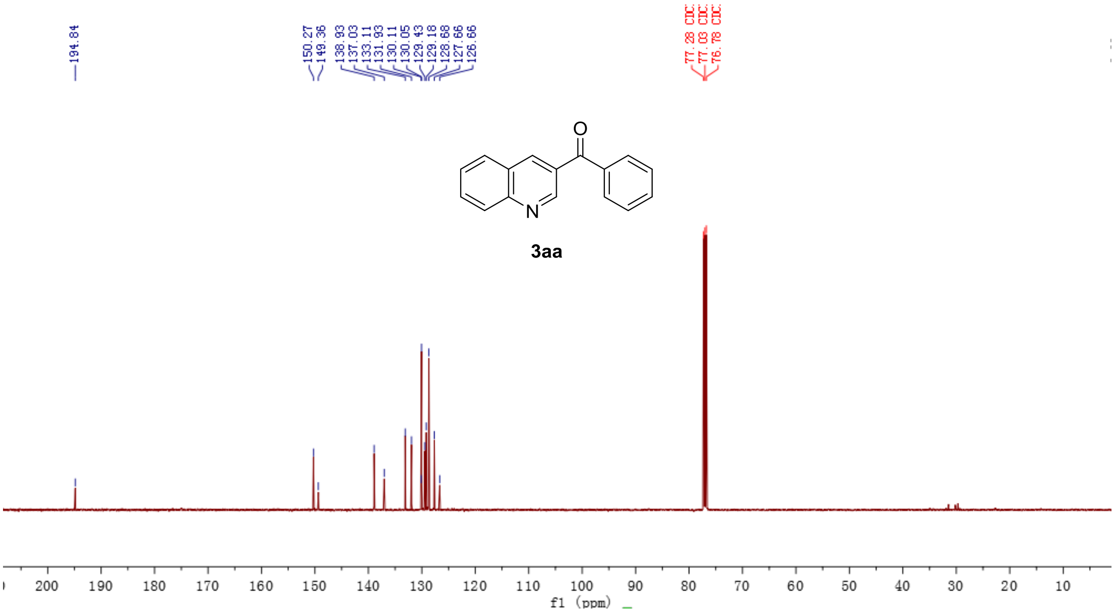
194.84
150.27
149.36
138.93
137.03
133.11
131.93
130.11
130.05
129.43
129.18
128.68
127.66
126.66
77.28 CDC
77.03 CDC
76.78 CDC
O
N
3aa
f1 (ppm)

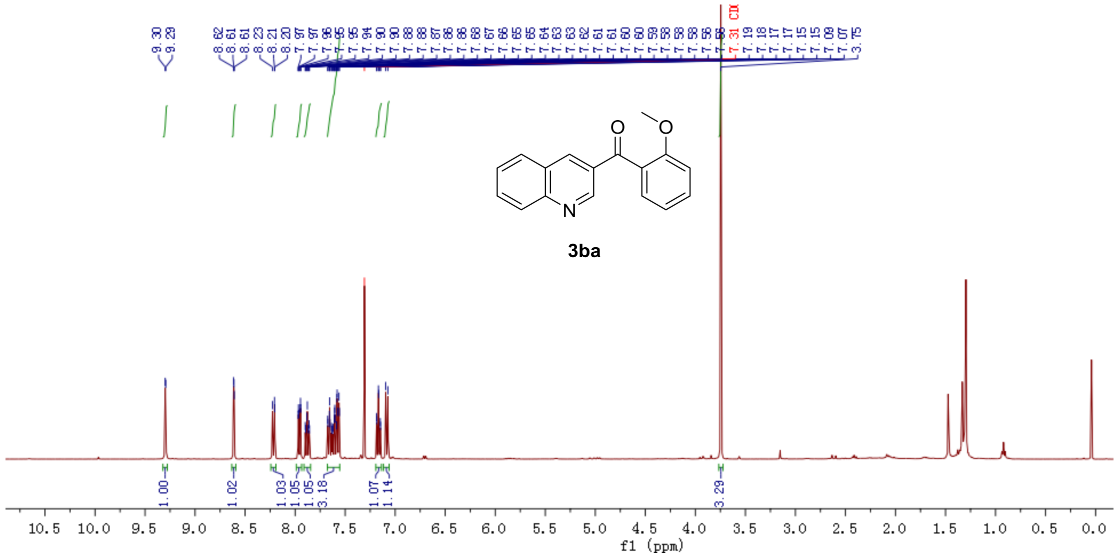
9.30
9.29
8.62
8.61
8.61
8.23
8.21
8.20
7.97
7.97
7.96
7.95
7.95
7.94
7.90
7.90
7.88
7.88
7.87
7.86
7.86
7.68
7.67
7.66
7.65
7.65
7.64
7.63
7.63
7.62
7.61
7.61
7.60
7.60
7.59
7.58
7.58
7.58
7.56
7.31 CDCl3
7.19
7.18
7.17
7.17
7.15
7.09
7.07
3.75
O
O
N
3ba
1.00
1.02
1.03
1.05
1.05
3.18
1.07
1.14
3.29
f1 (ppm)

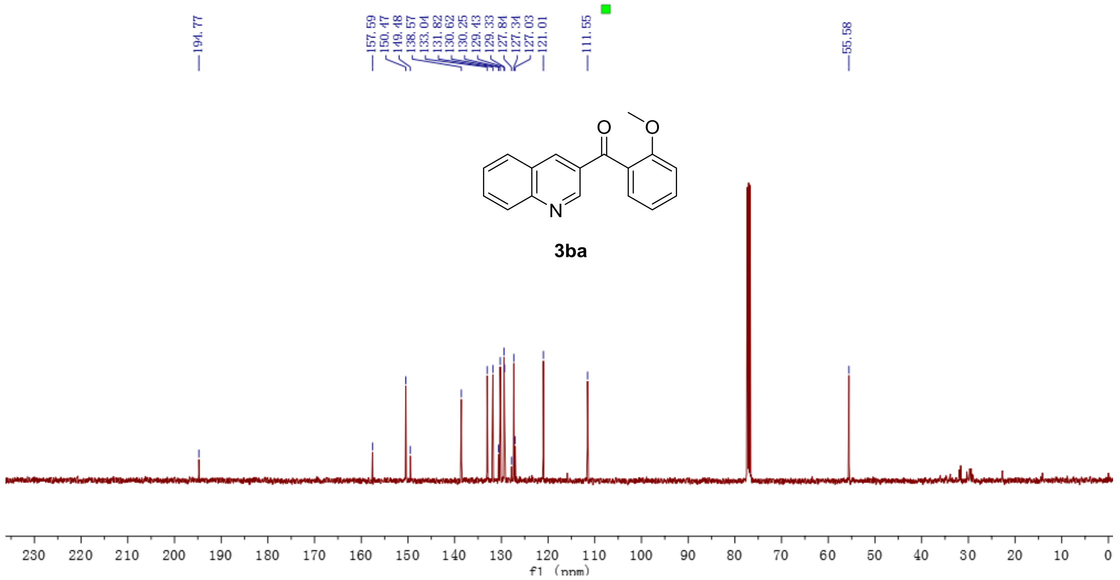
194.77
157.59
150.47
149.48
138.57
133.04
131.82
130.62
130.25
129.43
129.33
127.84
127.34
127.03
121.01
111.55
55.58
O
O
N
3ba
f1 (ppm)

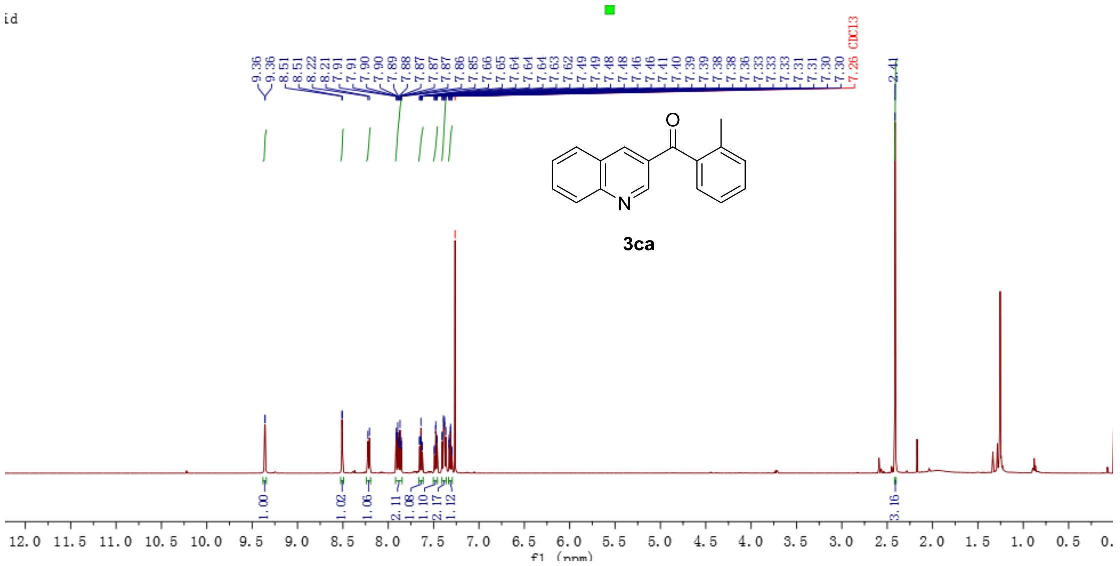

9.36
9.36
8.51
8.51
8.22
8.21
7.91
7.91
7.90
7.90
7.89
7.88
7.87
7.87
7.87
7.86
7.85
7.66
7.65
7.64
7.64
7.63
7.62
7.49
7.48
7.48
7.46
7.41
7.40
7.39
7.39
7.38
7.38
7.36
7.33
7.33
7.33
7.31
7.31
7.30
7.26 CDCl3
2.41
O
N
3ca
1.00
1.02
1.06
2.11
1.08
1.10
2.17
1.12
3.16
12.0 11.5 11.0 10.5 10.0 9.5 9.0 8.5 8.0 7.5 7.0 6.5 6.0 5.5 5.0 4.5 4.0 3.5 3.0 2.5 2.0 1.5 1.0 0.5 0.
f1 (ppm)

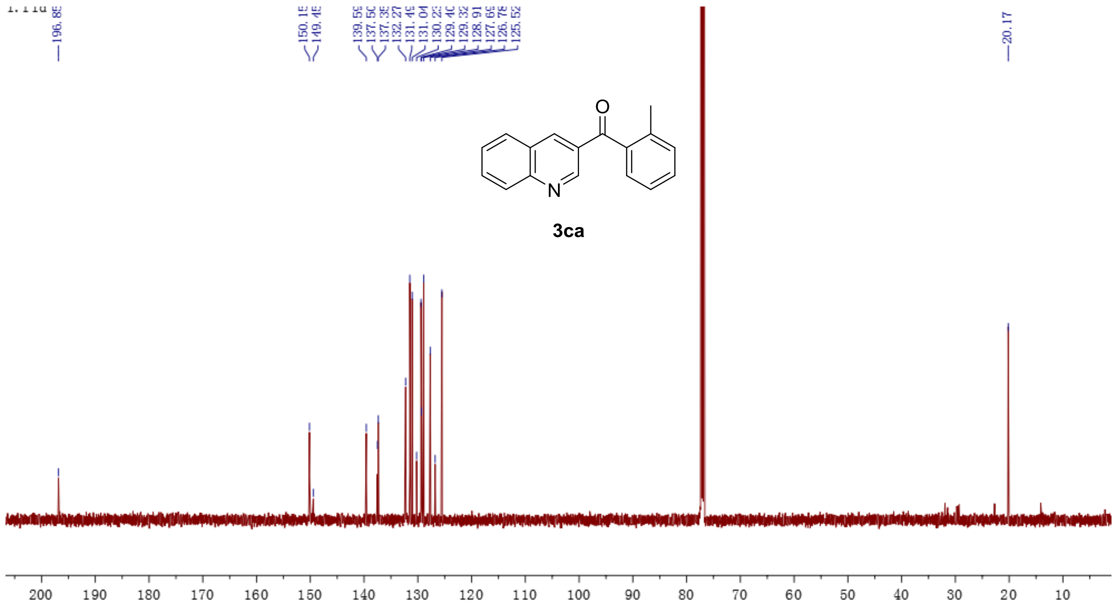

196.89
150.15
149.45
139.59
137.50
137.35
132.27
131.49
131.04
130.23
129.40
129.32
128.91
127.69
126.78
125.52
20.17
O
N
3ca
200 190 180 170 160 150 140 130 120 110 100 90 80 70 60 50 40 30 20 10

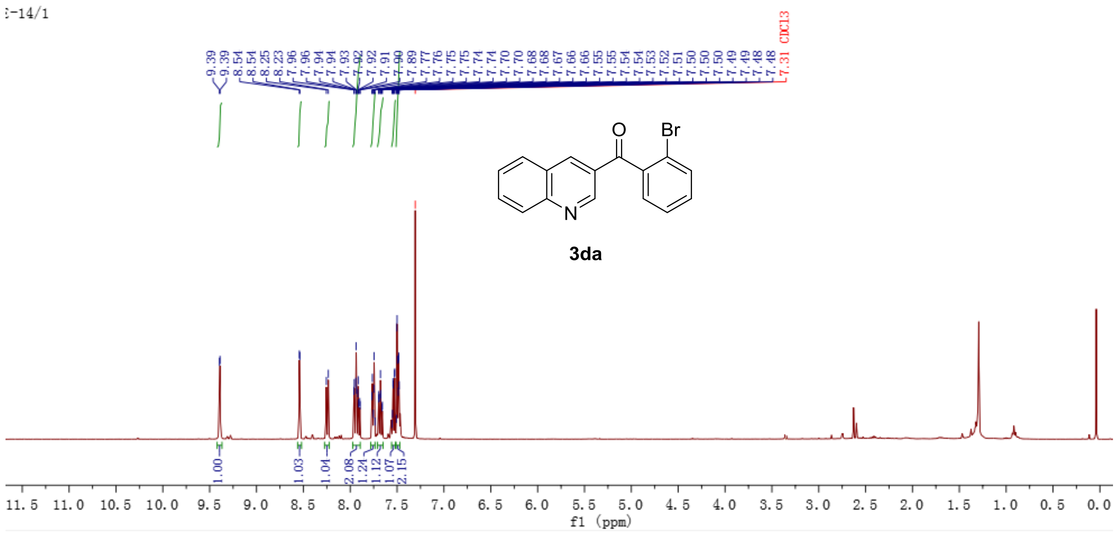

:-14/1
9.39
9.39
8.54
8.54
8.25
8.23
7.96
7.94
7.93
7.92
7.92
7.91
7.90
7.89
7.77
7.76
7.75
7.75
7.74
7.74
7.70
7.70
7.68
7.68
7.67
7.66
7.66
7.55
7.55
7.54
7.54
7.53
7.52
7.51
7.50
7.50
7.49
7.48
7.48
7.31 CDCl3
O
Br
N
3da
1.00
1.03
1.04
2.08
1.24
1.12
1.07
2.15
11.5 11.0 10.5 10.0 9.5 9.0 8.5 8.0 7.5 7.0 6.5 6.0 5.5 5.0 4.5 4.0 3.5 3.0 2.5 2.0 1.5 1.0 0.5 0.0
f1 (ppm)

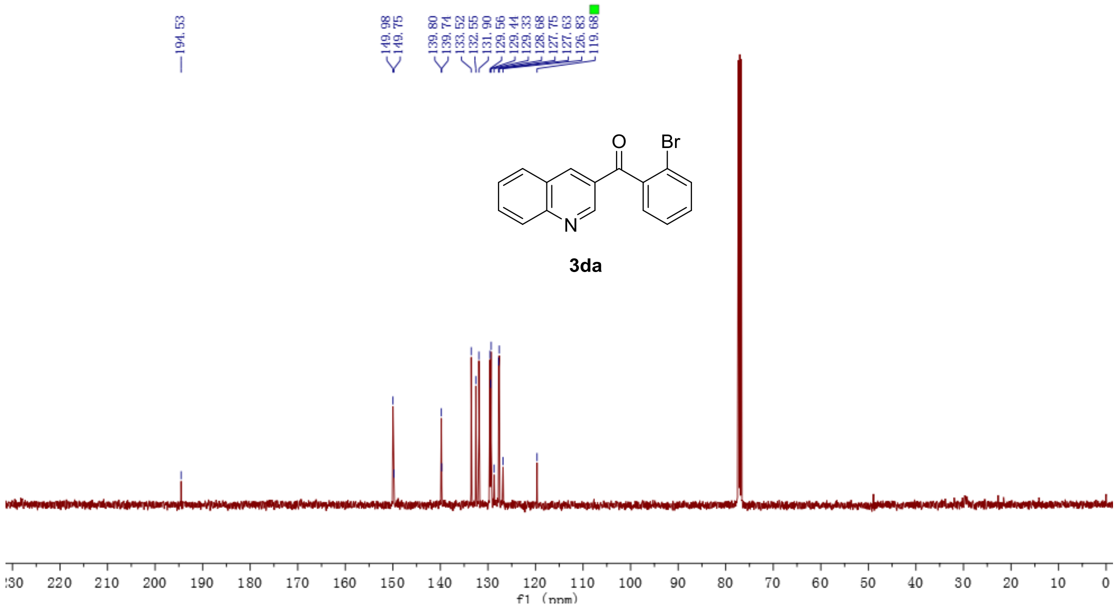

194.53
149.98
149.75
139.80
139.74
133.52
132.55
131.90
129.56
129.44
129.33
128.68
127.75
127.63
126.83
119.68
O
Br
N
3da
230 220 210 200 190 180 170 160 150 140 130 120 110 100 90 80 70 60 50 40 30 20 10 0
f1 (ppm)

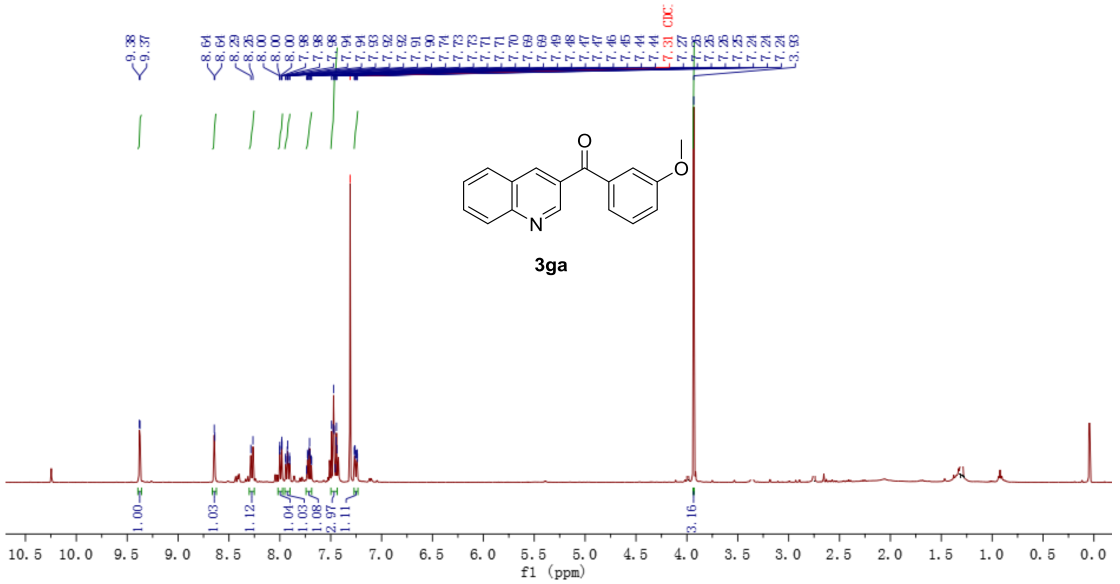
9.38
9.37
8.64
8.64
8.29
8.26
8.00
8.00
7.98
7.98
7.98
7.94
7.94
7.93
7.92
7.92
7.91
7.90
7.74
7.73
7.73
7.71
7.71
7.70
7.69
7.69
7.49
7.48
7.47
7.47
7.46
7.45
7.44
7.44
7.31 CDC
7.27
7.26
7.26
7.26
7.25
7.25
7.24
7.24
7.24
3.93
O
O
N
3ga
1.00
1.03
1.12
1.04
1.03
1.08
2.97
1.11
3.16
f1 (ppm)

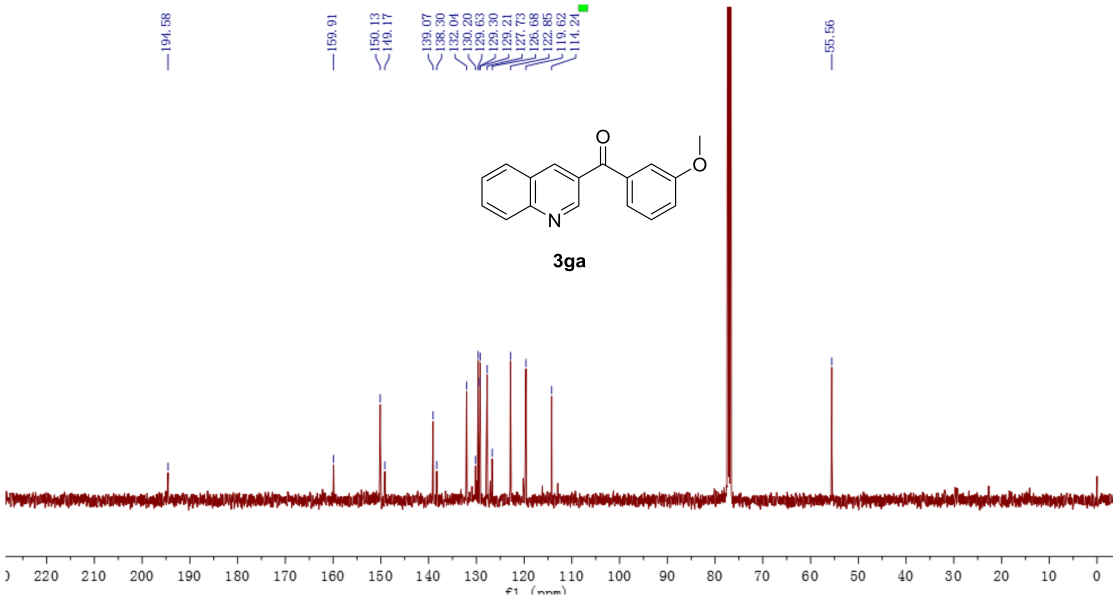
194.58
159.91
150.13
149.17
139.07
138.30
132.04
130.20
129.63
129.30
129.21
127.73
126.68
122.85
119.62
114.24
55.56
O
O
N
3ga
f1 (ppm)

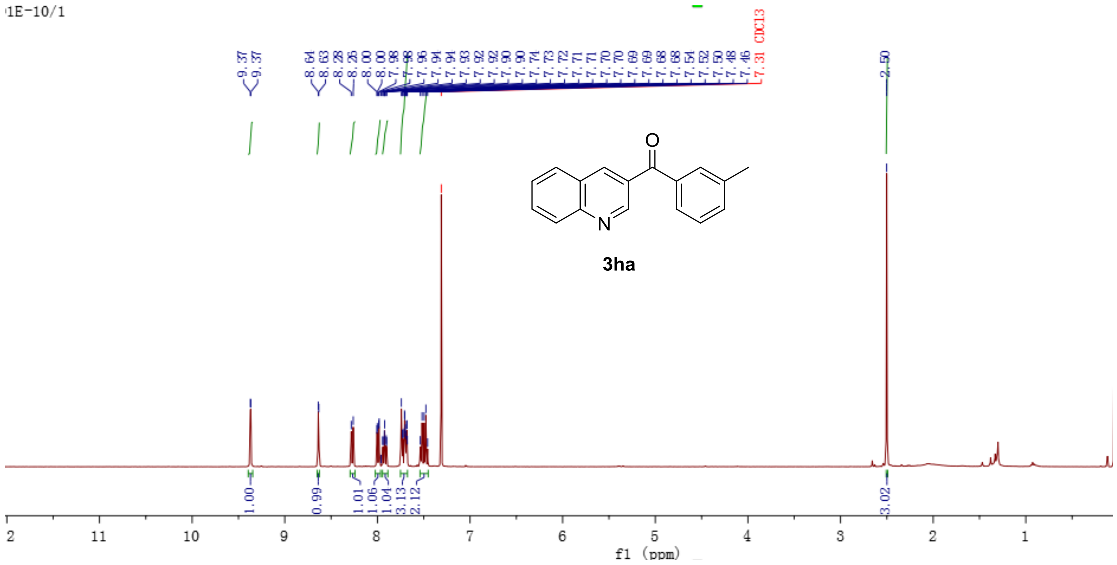
1E-10/1
9.37
9.37
8.64
8.63
8.28
8.26
8.00
8.00
7.98
7.96
7.94
7.94
7.93
7.92
7.92
7.90
7.74
7.73
7.72
7.71
7.71
7.70
7.70
7.69
7.69
7.68
7.68
7.54
7.52
7.50
7.48
7.46
7.31 CDCl3
2.50
O
N
3ha
1.00
0.99
1.01
1.06
1.04
3.13
2.12
3.02
f1 (ppm)

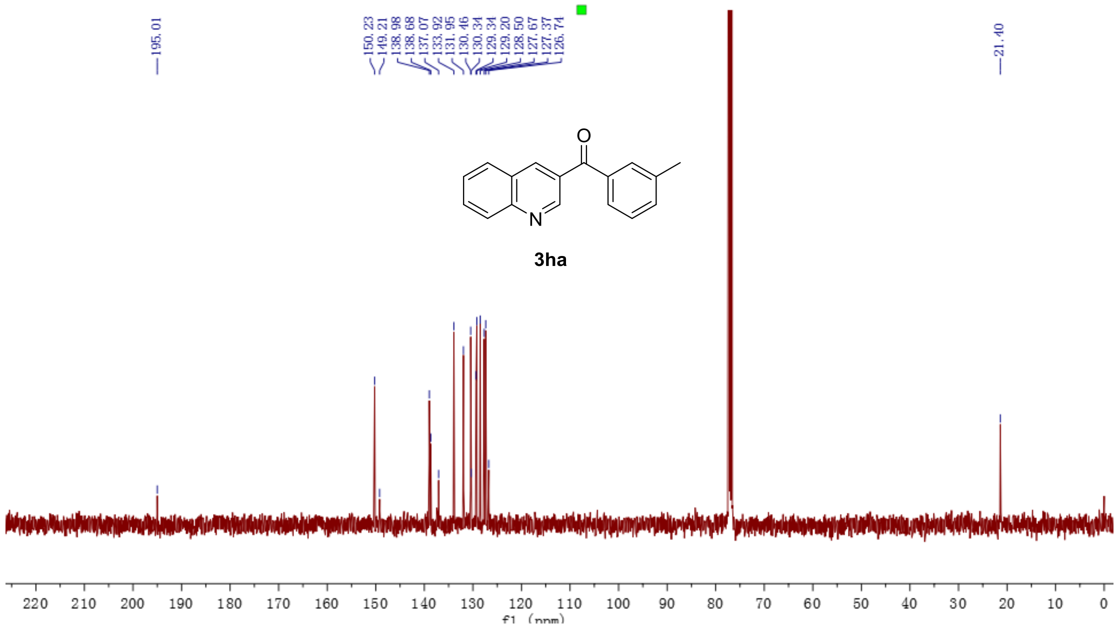
195.01
150.23
149.21
138.98
138.68
137.07
133.92
131.95
130.46
130.34
129.34
129.20
128.50
127.67
127.37
126.74
21.40
O
N
3ha
f1 (ppm)

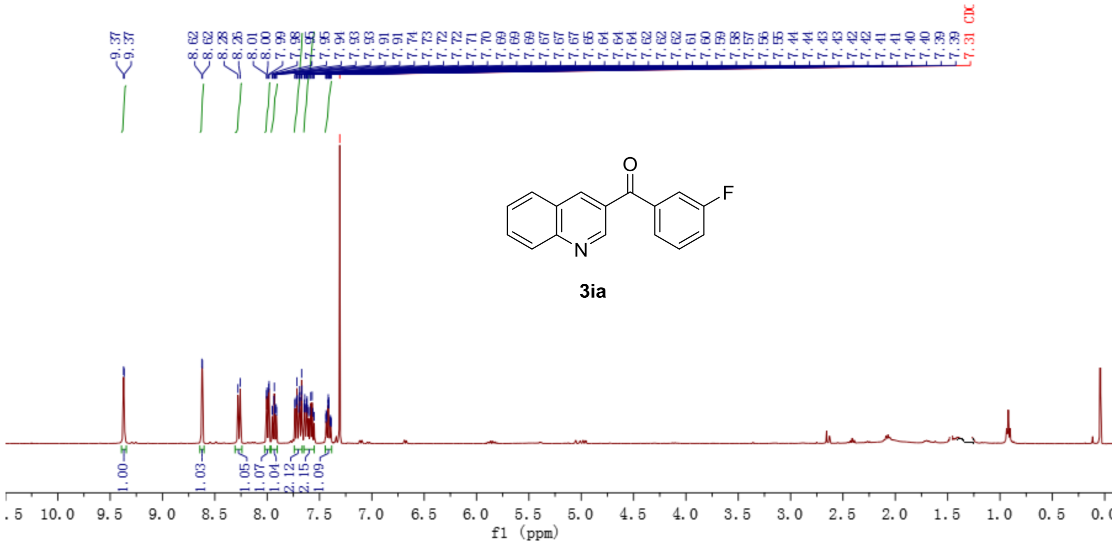
3ia
f1 (ppm)
9.37
9.37
8.62
8.62
8.28
8.25
8.01
8.00
7.31 CDC
1.00
1.03
1.05
1.07
1.04
2.12
2.15
1.09

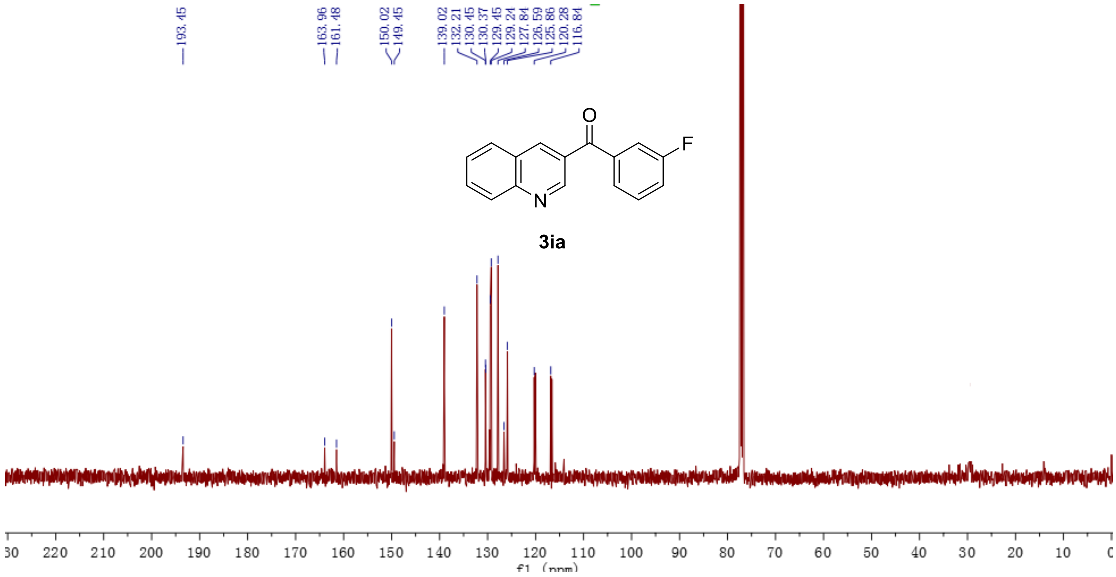
3ia
193.45
163.96
161.48
150.02
149.45
139.02
132.21
130.45
130.37
129.45
129.24
127.84
126.59
125.86
120.28
116.84
f1 (ppm)

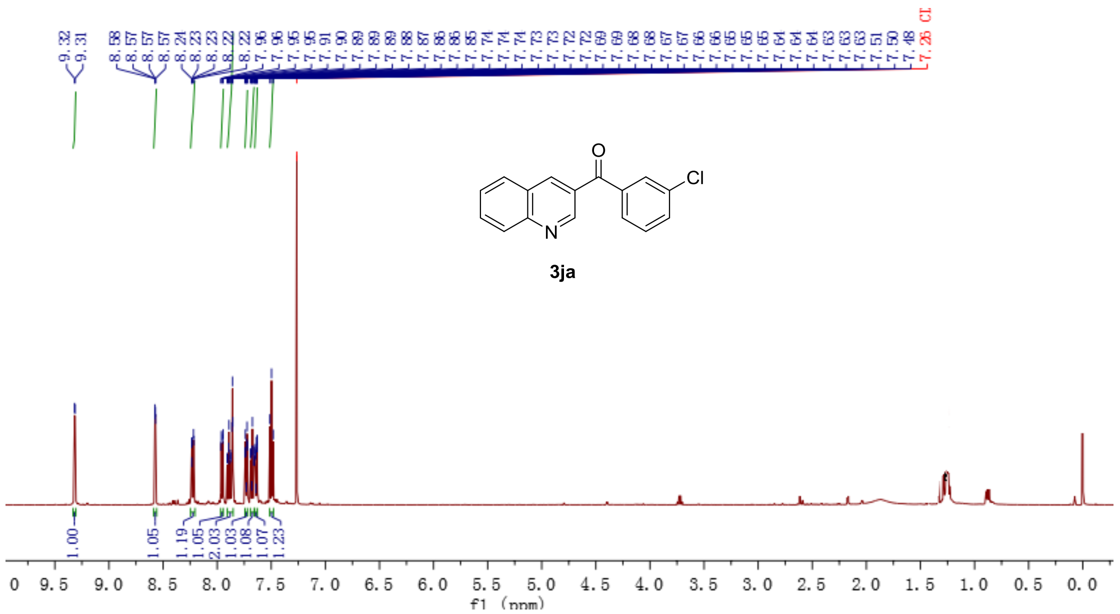
9.32
9.31
8.58
8.57
8.57
8.57
8.24
8.23
8.23
8.22
8.22
7.96
7.96
7.95
7.95
7.91
7.90
7.89
7.89
7.89
7.88
7.87
7.86
7.86
7.85
7.74
7.74
7.74
7.73
7.73
7.72
7.72
7.69
7.69
7.68
7.68
7.67
7.67
7.66
7.66
7.65
7.65
7.65
7.64
7.64
7.64
7.63
7.63
7.63
7.51
7.50
7.48
7.26 CDCl3
O
Cl
N
3ja
1.00
1.05
1.19
1.05
2.03
1.03
1.08
1.07
1.23
f1 (ppm)

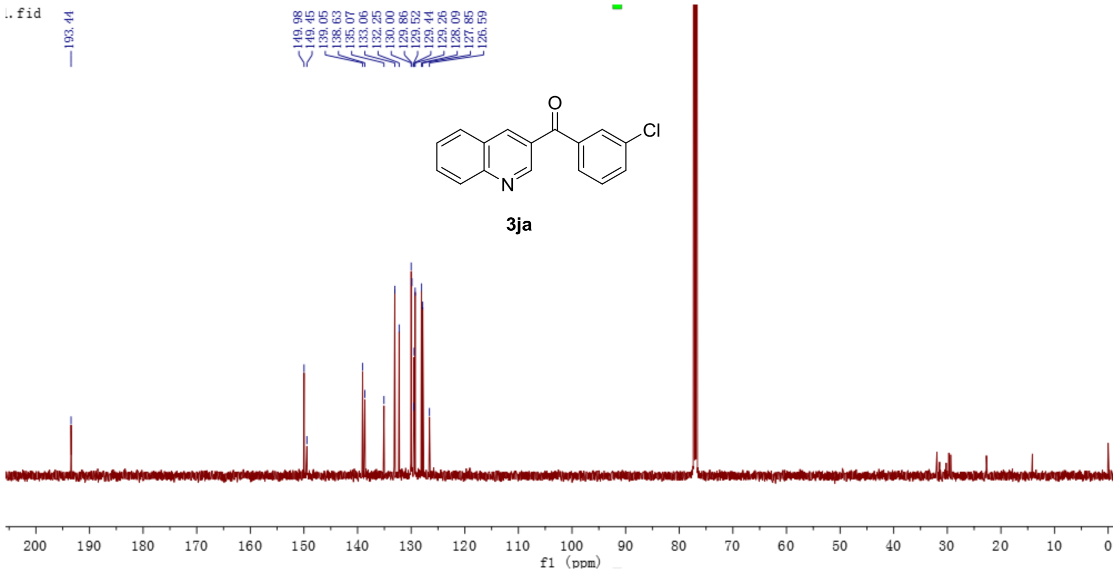
193.44
149.98
149.45
139.05
138.63
135.07
133.06
132.25
130.00
129.86
129.52
129.44
129.26
128.09
127.85
126.59
O
Cl
N
3ja
f1 (ppm)

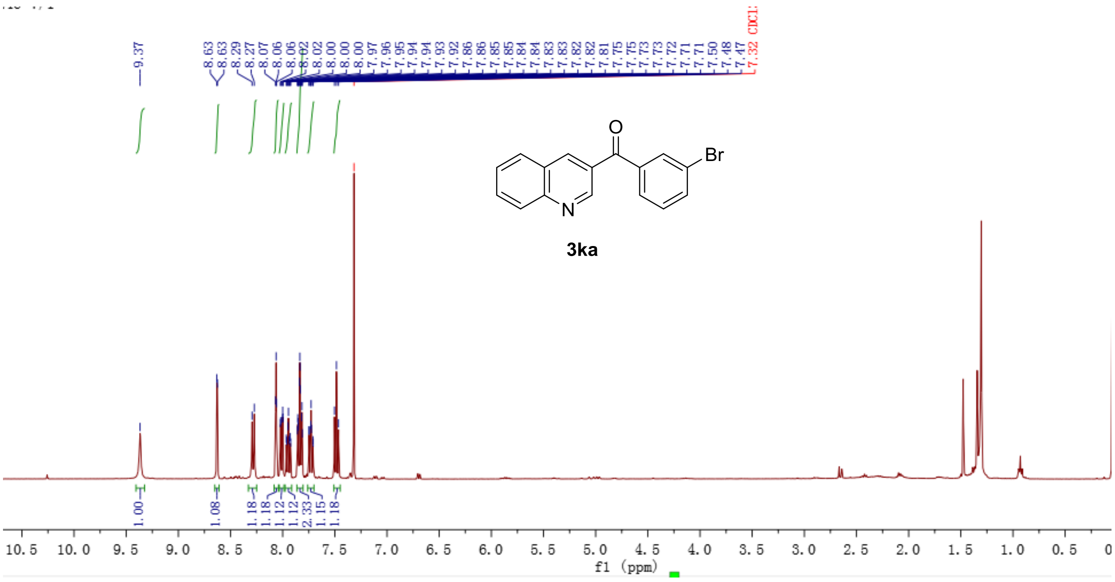
9.37
8.63
8.63
8.29
8.27
8.07
8.06
8.06
8.00
7.97
7.96
7.95
7.94
7.94
7.93
7.92
7.86
7.86
7.85
7.85
7.84
7.83
7.83
7.82
7.82
7.81
7.75
7.75
7.73
7.73
7.72
7.71
7.71
7.50
7.48
7.47
7.32 CDCl3
O
Br
N
3ka
1.00
1.08
1.18
1.18
1.12
1.12
2.33
1.15
1.18
10.5
10.0
9.5
9.0
8.5
8.0
7.5
7.0
6.5
6.0
5.5
5.0
4.5
4.0
3.5
3.0
2.5
2.0
1.5
1.0
0.5
0
f1 (ppm)

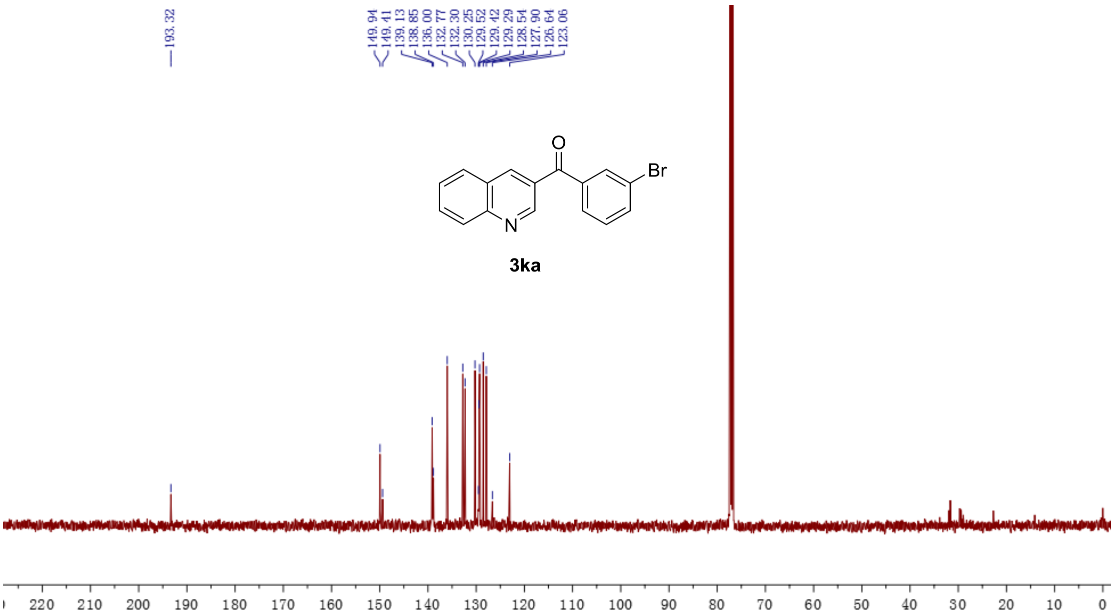
193.32
149.94
149.41
139.13
138.85
136.00
132.77
132.30
130.25
129.52
129.42
129.29
128.54
127.90
126.64
123.06
O
Br
N
3ka
220
210
200
190
180
170
160
150
140
130
120
110
100
90
80
70
60
50
40
30
20
10
0

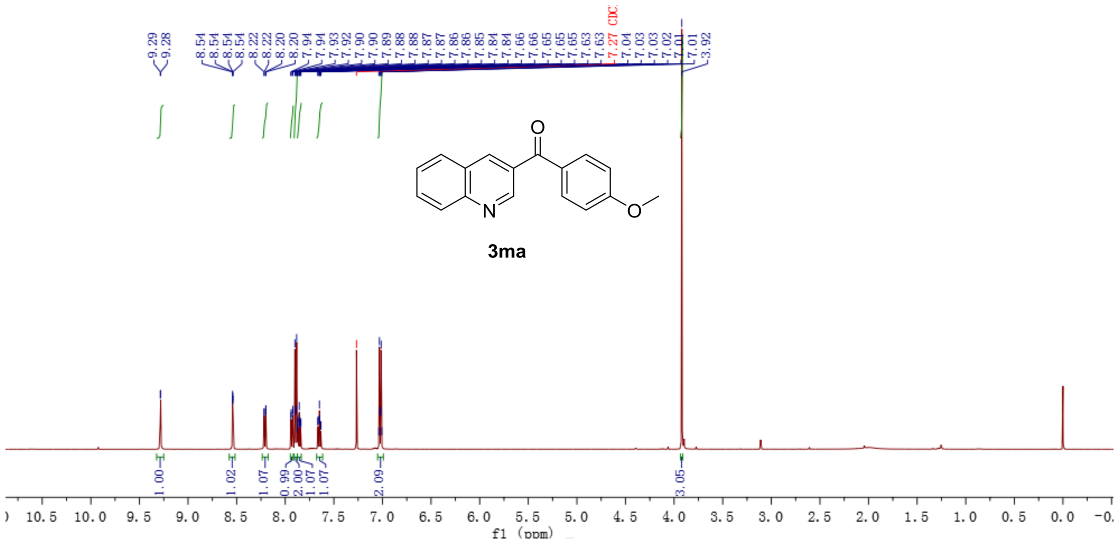
3ma
f1 (ppm)
9.29
9.28
3.92
1.00
1.02
1.07
0.99
2.00
1.07
1.07
2.09
3.05

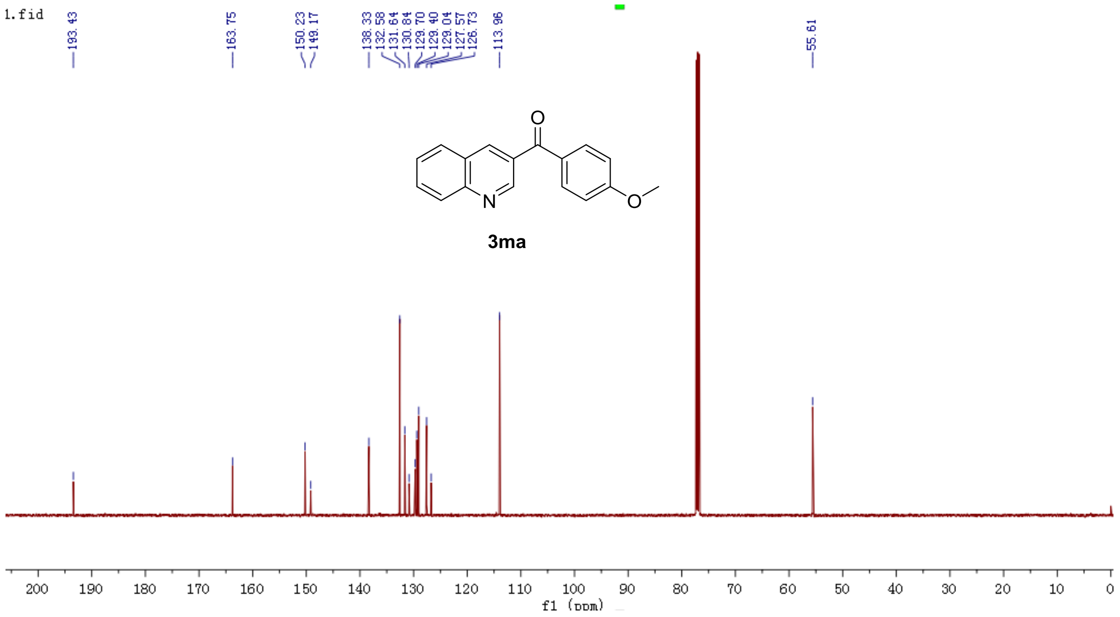
3ma
f1 (ppm)
193.43
163.75
150.23
149.17
138.33
132.58
131.64
130.84
129.70
129.40
129.04
127.57
126.73
113.96
55.61

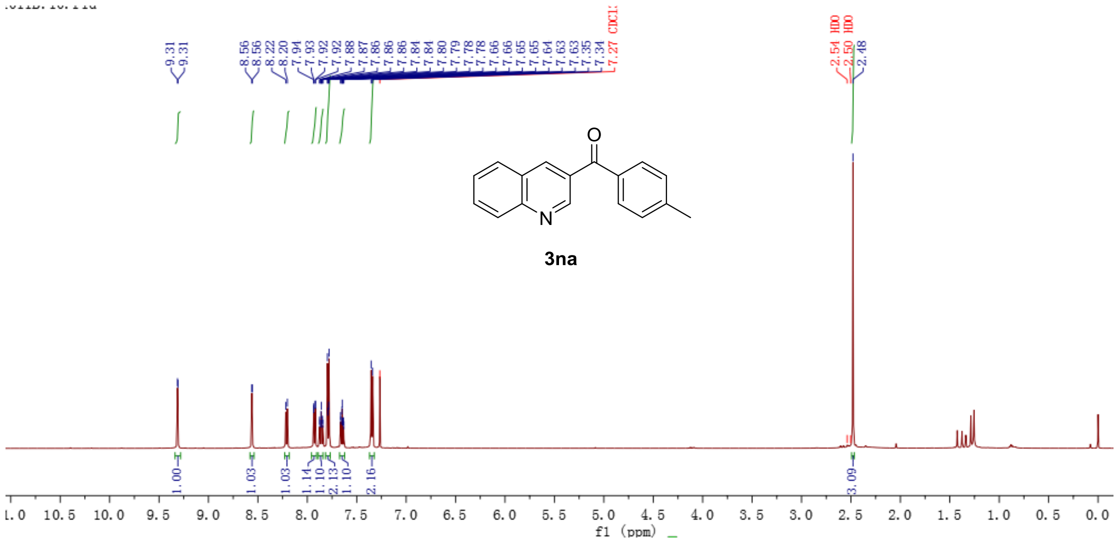
9.31
9.31
8.56
8.56
8.22
8.20
7.94
7.93
7.92
7.92
7.88
7.87
7.86
7.86
7.84
7.84
7.80
7.79
7.78
7.78
7.66
7.66
7.65
7.65
7.64
7.63
7.63
7.35
7.34
7.27 CDCl3
2.54 HDO
2.50 HDO
2.48
O
N
3na
1.00
1.03
1.03
1.14
1.10
2.13
1.10
2.16
3.09
f1 (ppm)

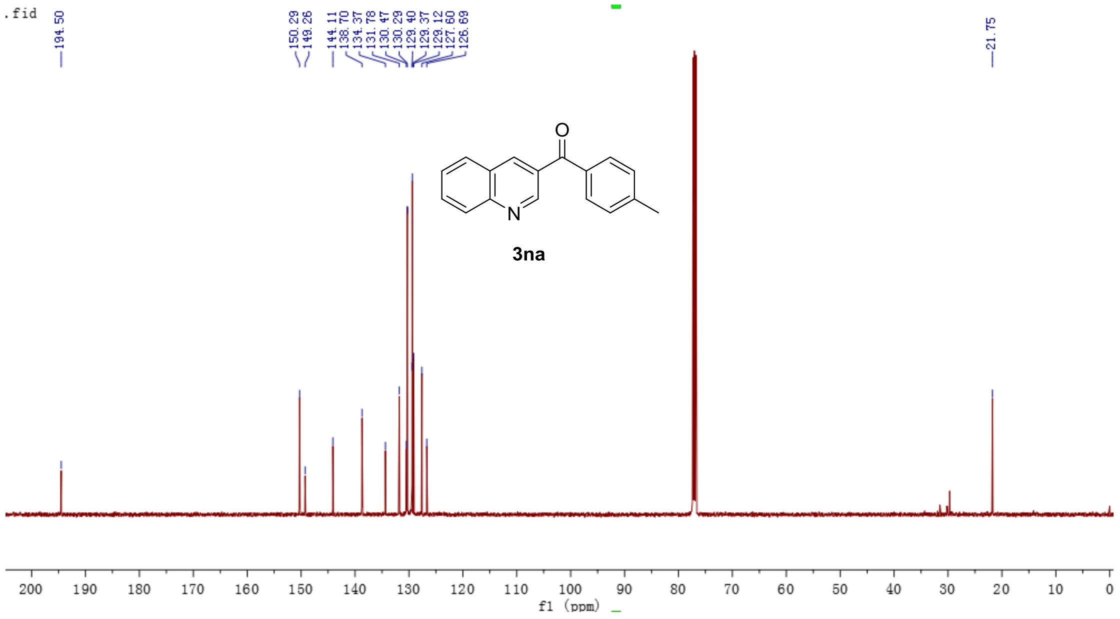
194.50
150.29
149.26
144.11
138.70
134.37
131.78
130.47
130.29
129.40
129.37
129.12
127.60
126.69
21.75
O
N
3na
f1 (ppm)

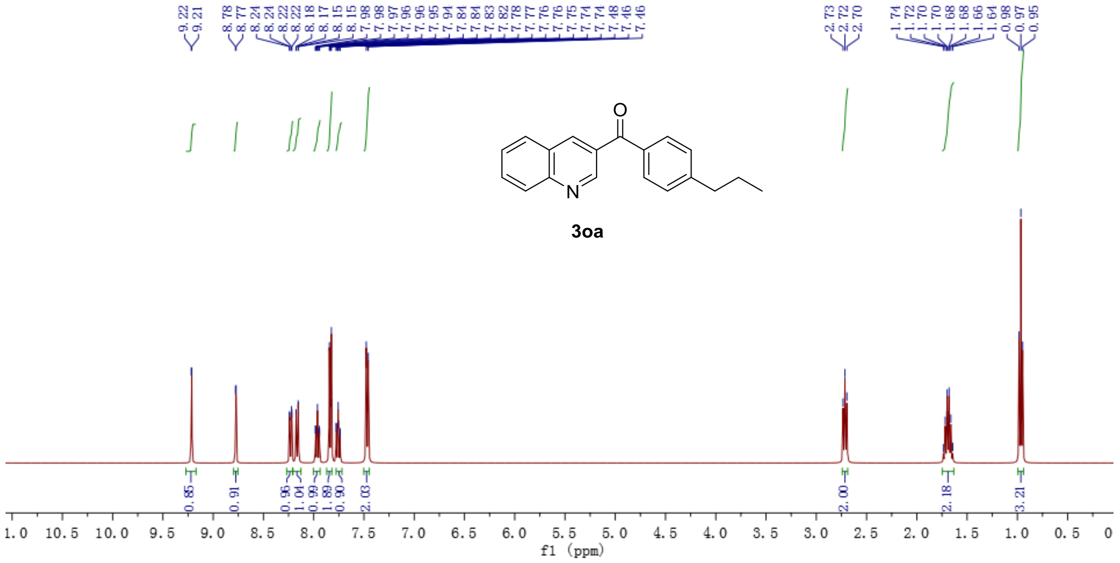
9.22
9.21
8.78
8.77
8.24
8.24
8.22
8.22
8.18
8.17
8.15
7.98
7.97
7.96
7.96
7.95
7.94
7.84
7.84
7.83
7.82
7.78
7.77
7.76
7.75
7.74
7.74
7.48
7.46
7.46
2.73
2.72
2.70
1.74
1.72
1.70
1.70
1.68
1.68
1.66
1.64
0.98
0.97
0.95
O
N
3oa
0.85
0.91
0.96
1.04
0.99
1.89
0.90
2.03
2.00
2.18
3.21
f1 (ppm)

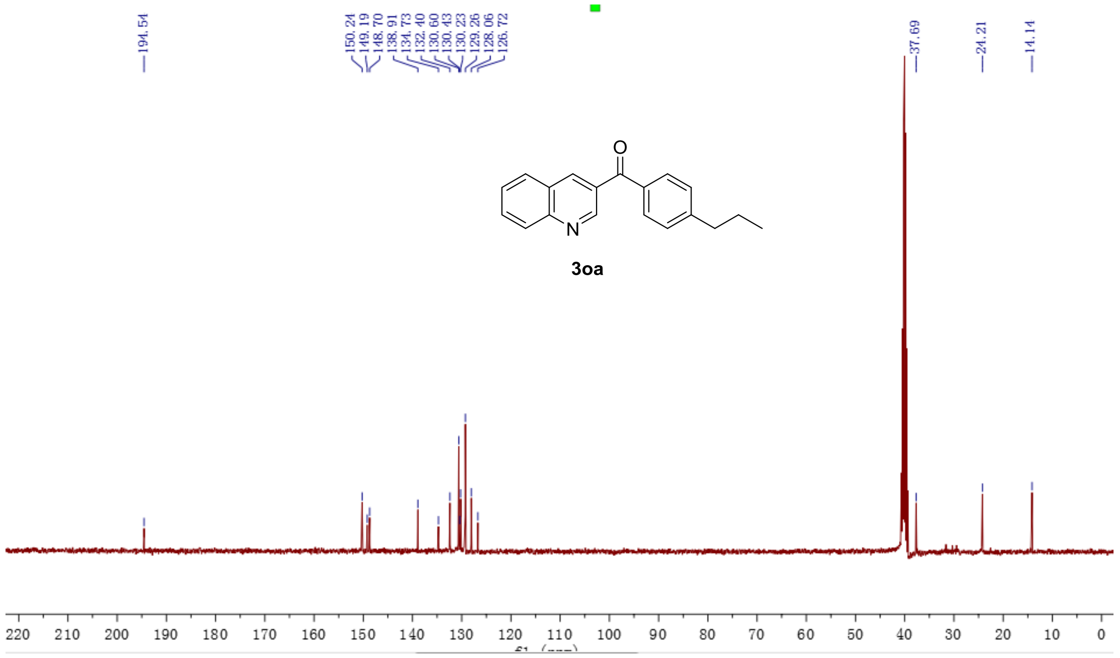
194.54
150.24
149.19
148.70
138.91
134.73
132.40
130.60
130.43
130.23
129.26
128.06
126.72
37.69
24.21
14.14
O
N
3oa

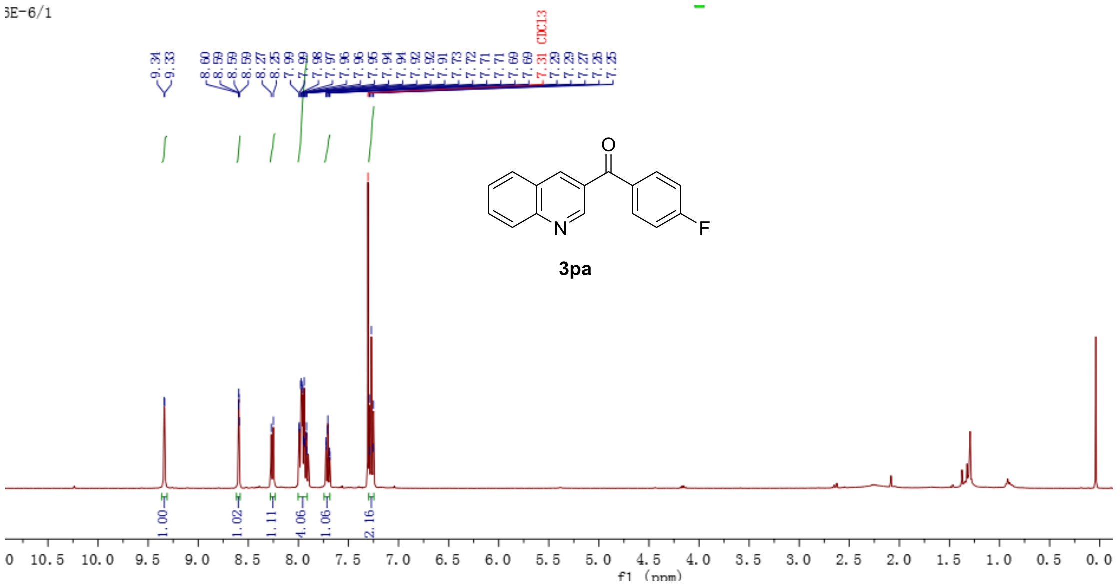

9.34
9.33
8.60
8.59
8.59
8.59
8.27
8.25
7.99
7.98
7.97
7.96
7.96
7.95
7.94
7.94
7.92
7.92
7.91
7.73
7.72
7.71
7.71
7.69
7.69
7.31 CDCl3
7.29
7.29
7.27
7.26
7.25
O
N
F
3pa
1.00
1.02
1.11
4.06
1.06
2.16
10.5 10.0 9.5 9.0 8.5 8.0 7.5 7.0 6.5 6.0 5.5 5.0 4.5 4.0 3.5 3.0 2.5 2.0 1.5 1.0 0.5 0.0
f1 (ppm)

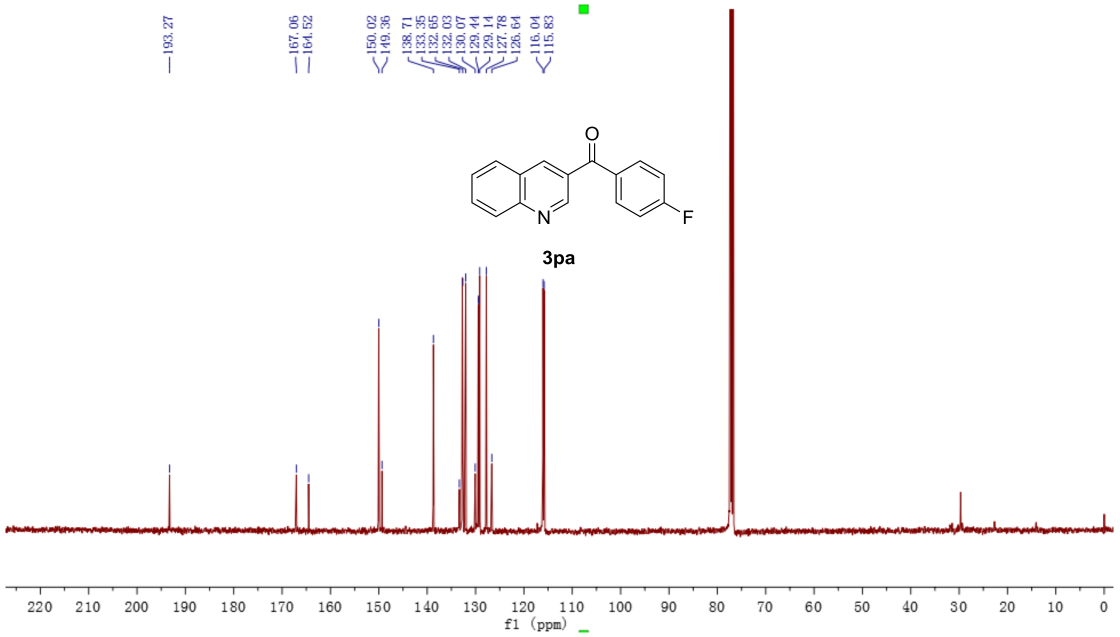

193.27
167.06
164.52
150.02
149.36
138.71
133.35
132.65
132.03
130.07
129.44
129.14
127.78
126.64
116.04
115.83
O
N
F
3pa
220 210 200 190 180 170 160 150 140 130 120 110 100 90 80 70 60 50 40 30 20 10 0
f1 (ppm)

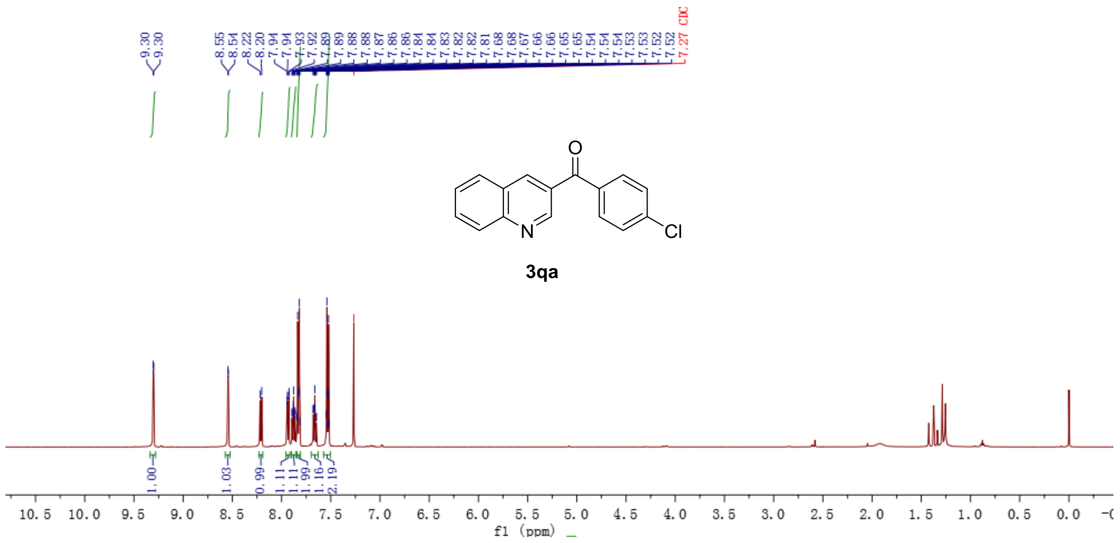
9.30
9.30
8.55
8.54
8.22
8.20
7.27 CDC
3qa
1.00
1.03
0.99
1.11
1.11
1.99
1.16
2.19
10.5
10.0
9.5
9.0
8.5
8.0
7.5
7.0
6.5
6.0
5.5
5.0
4.5
4.0
3.5
3.0
2.5
2.0
1.5
1.0
0.5
0.0
f1 (ppm)

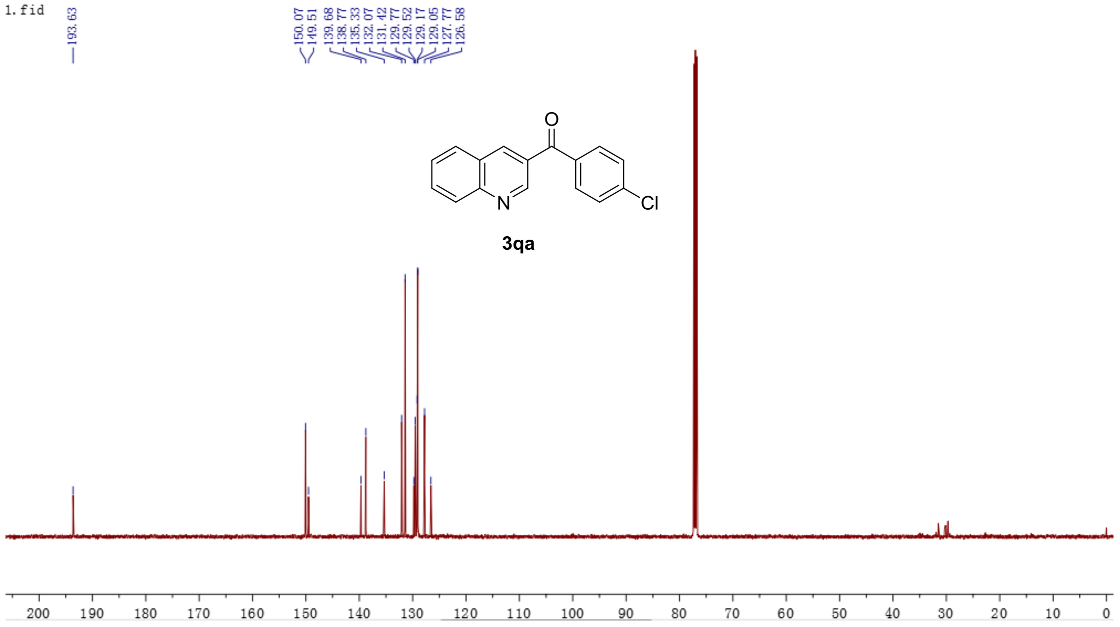
1.fid
193.63
150.07
149.51
139.68
138.77
135.33
132.07
131.42
129.77
129.52
129.17
129.05
127.77
126.58
3qa
200
190
180
170
160
150
140
130
120
110
100
90
80
70
60
50
40
30
20
10
0

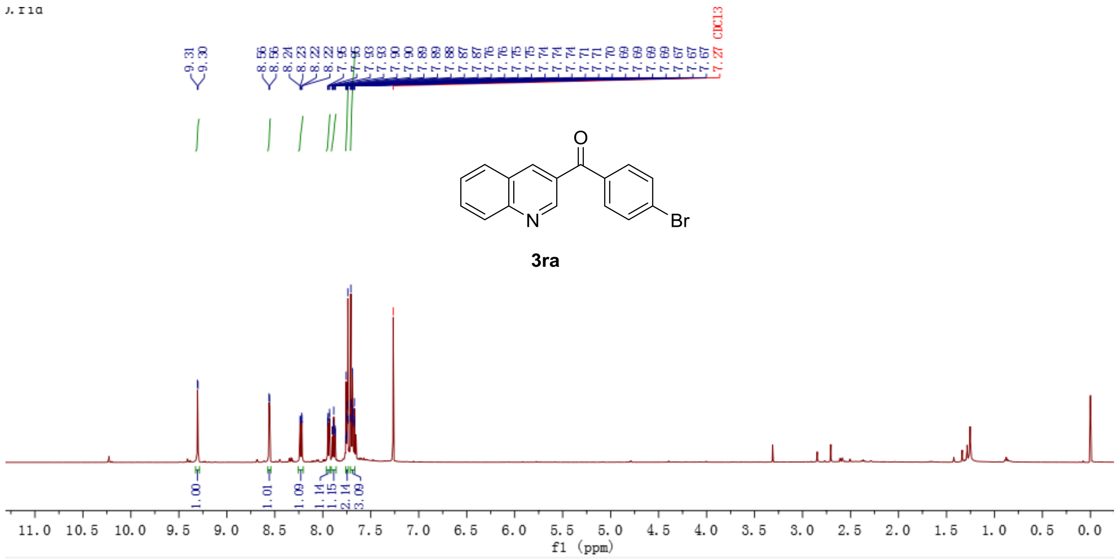
9.31
9.30
8.56
8.56
8.24
8.23
8.22
8.22
7.95
7.95
7.93
7.93
7.90
7.90
7.89
7.89
7.88
7.87
7.87
7.76
7.76
7.75
7.75
7.74
7.74
7.74
7.71
7.71
7.70
7.69
7.69
7.69
7.69
7.67
7.67
7.67
7.27 CDCl3
O
N
Br
3ra
1.00
1.01
1.09
1.14
1.15
2.14
3.09
11.0 10.5 10.0 9.5 9.0 8.5 8.0 7.5 7.0 6.5 6.0 5.5 5.0 4.5 4.0 3.5 3.0 2.5 2.0 1.5 1.0 0.5 0.0
f1 (ppm)

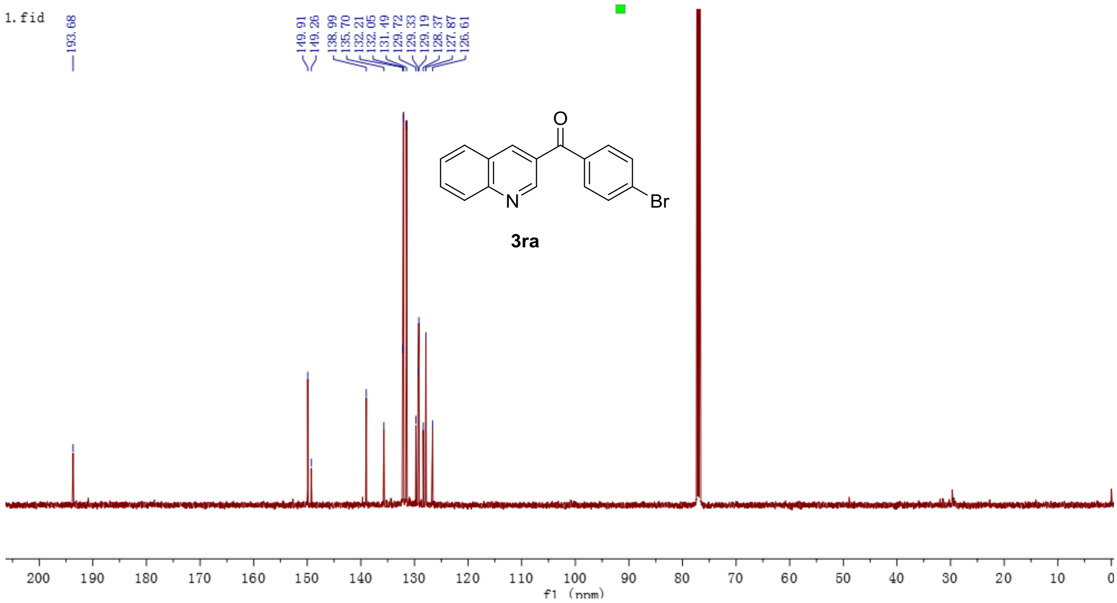
1.fid
193.68
149.91
149.26
138.99
135.70
132.21
132.05
131.49
129.72
129.33
129.19
128.37
127.87
126.61
O
N
Br
3ra
200 190 180 170 160 150 140 130 120 110 100 90 80 70 60 50 40 30 20 10 0
f1 (ppm)

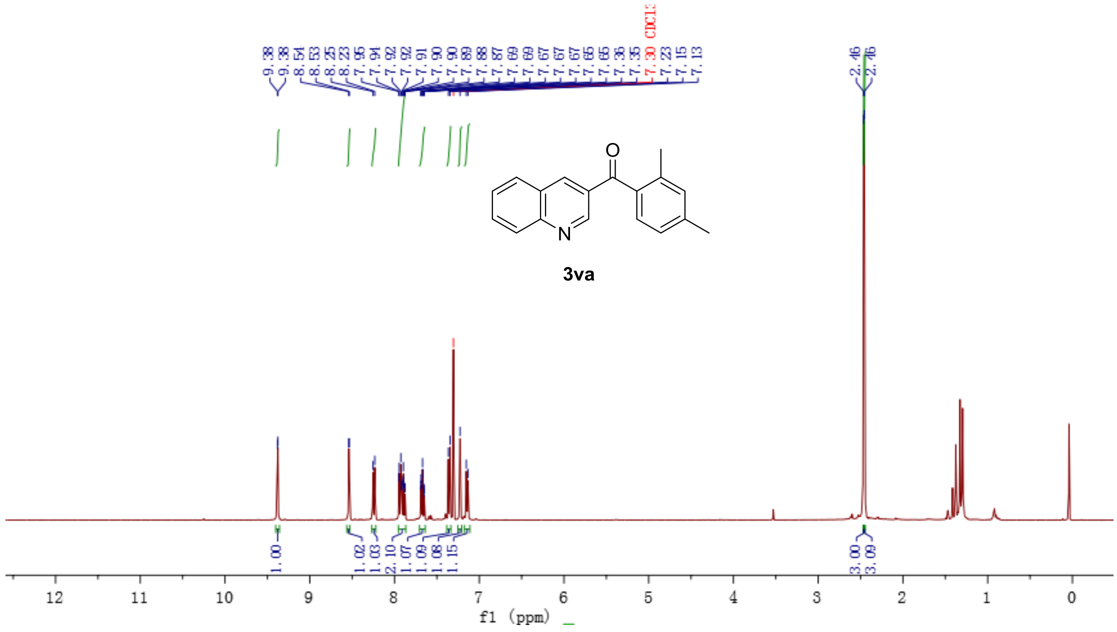
9.38
9.38
8.54
8.53
8.25
8.23
7.95
7.94
7.92
7.92
7.91
7.90
7.90
7.89
7.88
7.87
7.69
7.69
7.67
7.67
7.65
7.65
7.36
7.35
7.30 CDCl3
7.23
7.15
7.13
2.46
2.46
O
N
3va
1.00
1.02
1.03
2.10
1.07
1.09
1.08
1.15
3.00
3.09
f1 (ppm)

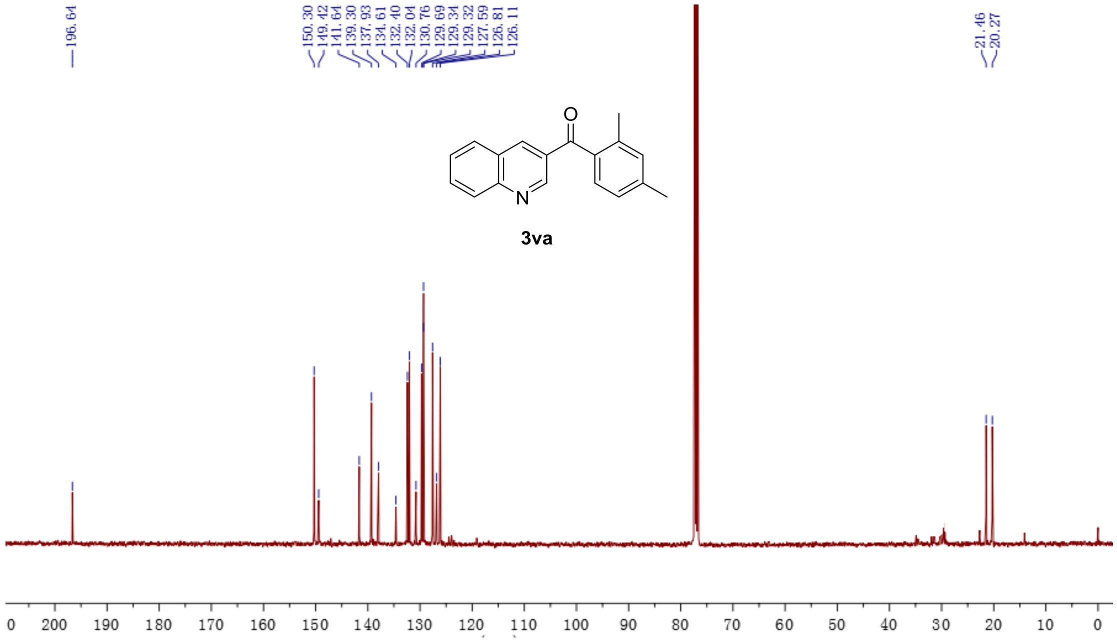
196.64
150.30
149.42
141.64
139.30
137.93
134.61
132.40
132.04
130.76
129.69
129.34
129.32
127.59
126.81
126.11
21.46
20.27
O
N
3va

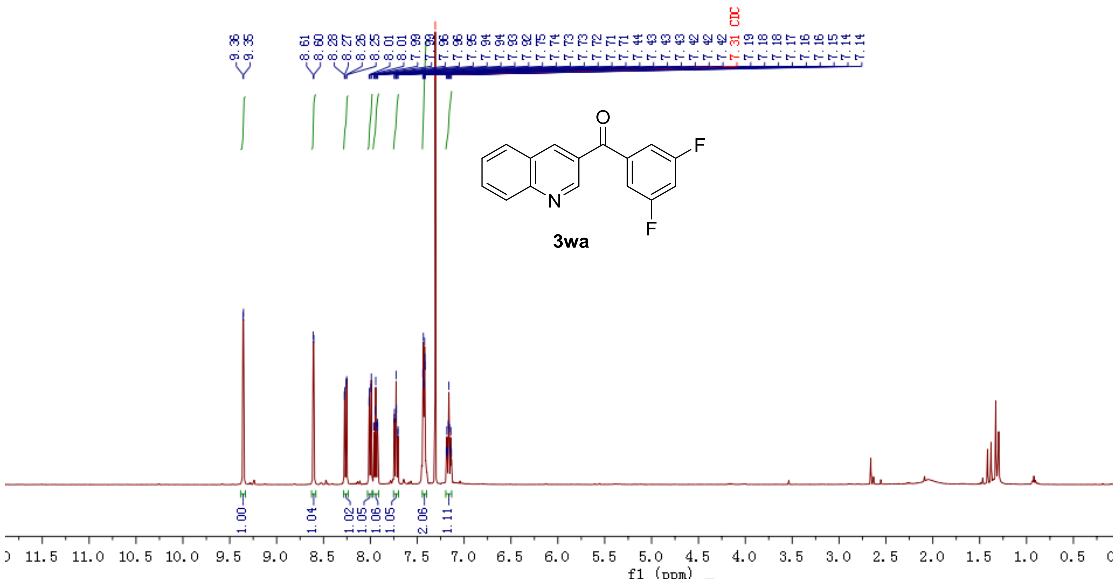
9.36
9.35
8.61
8.60
8.28
8.26
8.27
8.25
8.01
8.01
7.99
7.96
7.96
7.95
7.94
7.94
7.93
7.92
7.75
7.74
7.73
7.73
7.72
7.71
7.71
7.44
7.43
7.43
7.42
7.42
7.31 CDC
7.19
7.18
7.18
7.17
7.16
7.16
7.15
7.14
7.14
O
F
N
F
3wa
1.00
1.04
1.02
1.05
1.06
1.05
2.06
1.11
11.5 11.0 10.5 10.0 9.5 9.0 8.5 8.0 7.5 7.0 6.5 6.0 5.5 5.0 4.5 4.0 3.5 3.0 2.5 2.0 1.5 1.0 0.5
f1 (ppm)

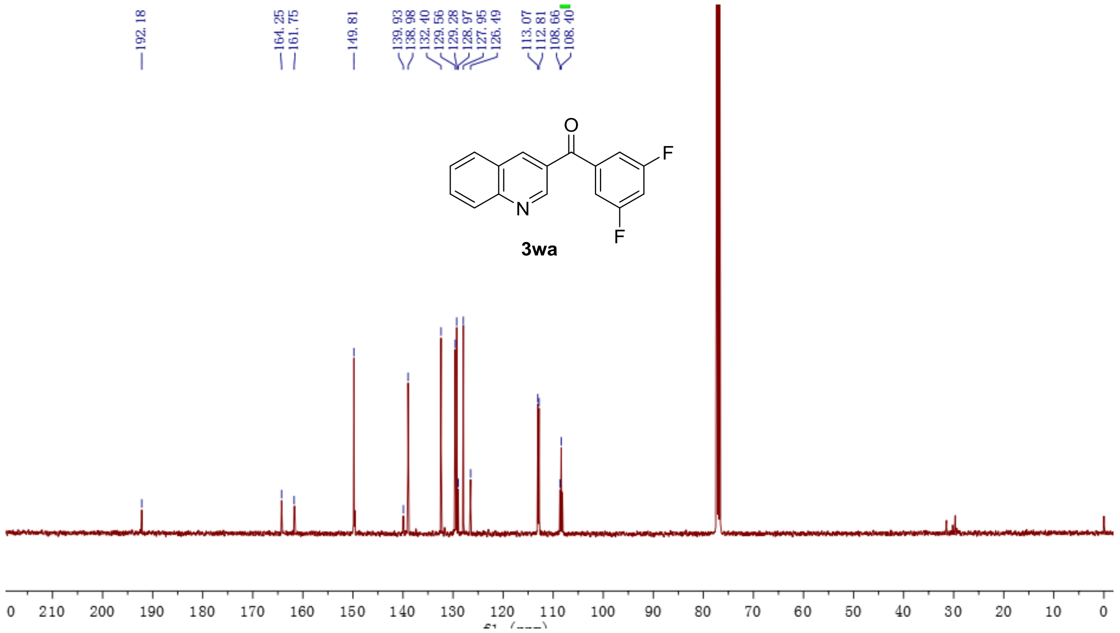
192.18
164.25
161.75
149.81
139.93
138.98
132.40
129.56
129.28
128.97
127.95
126.49
113.07
112.81
108.66
108.40
O
F
N
F
3wa
210 200 190 180 170 160 150 140 130 120 110 100 90 80 70 60 50 40 30 20 10 0

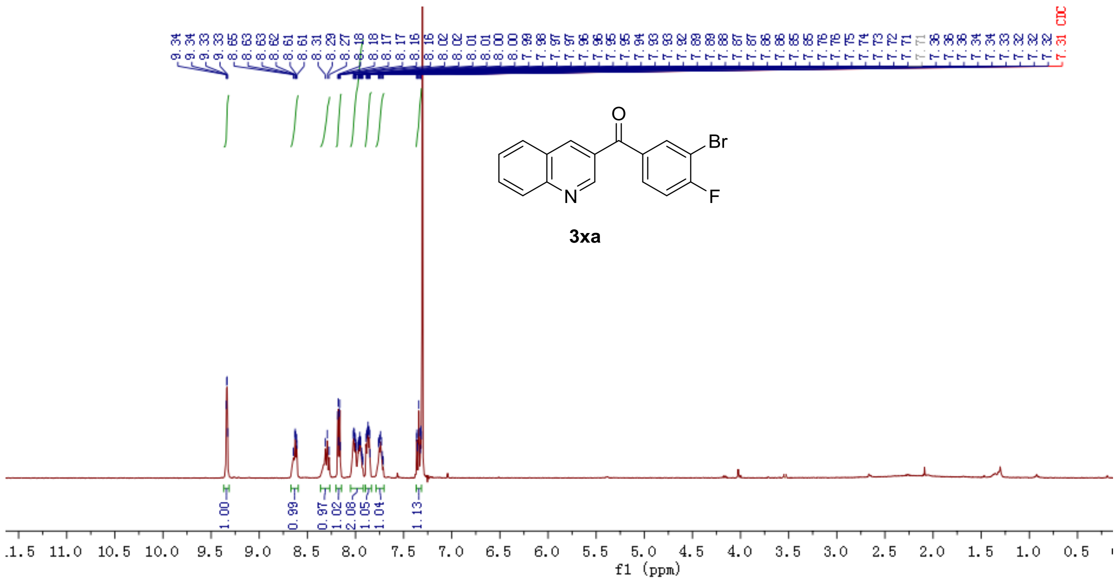

Br
F
N
O
3xa
f1 (ppm)

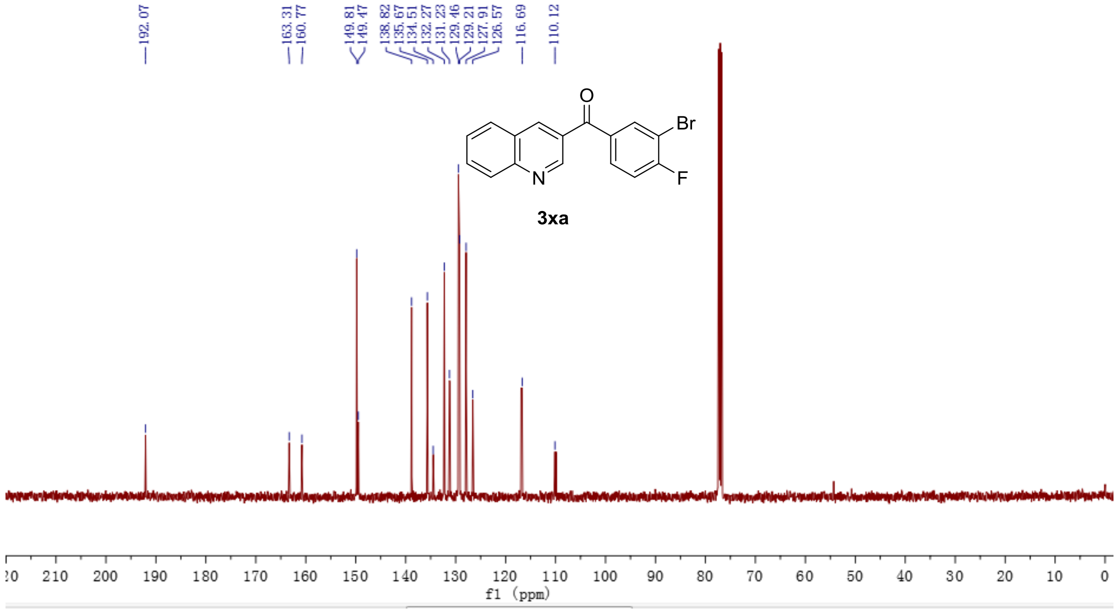

192.07
163.31
160.77
149.81
149.47
138.82
135.67
134.51
132.27
131.23
129.46
129.21
127.91
126.57
116.69
110.12
Br
F
N
O
3xa
f1 (ppm)

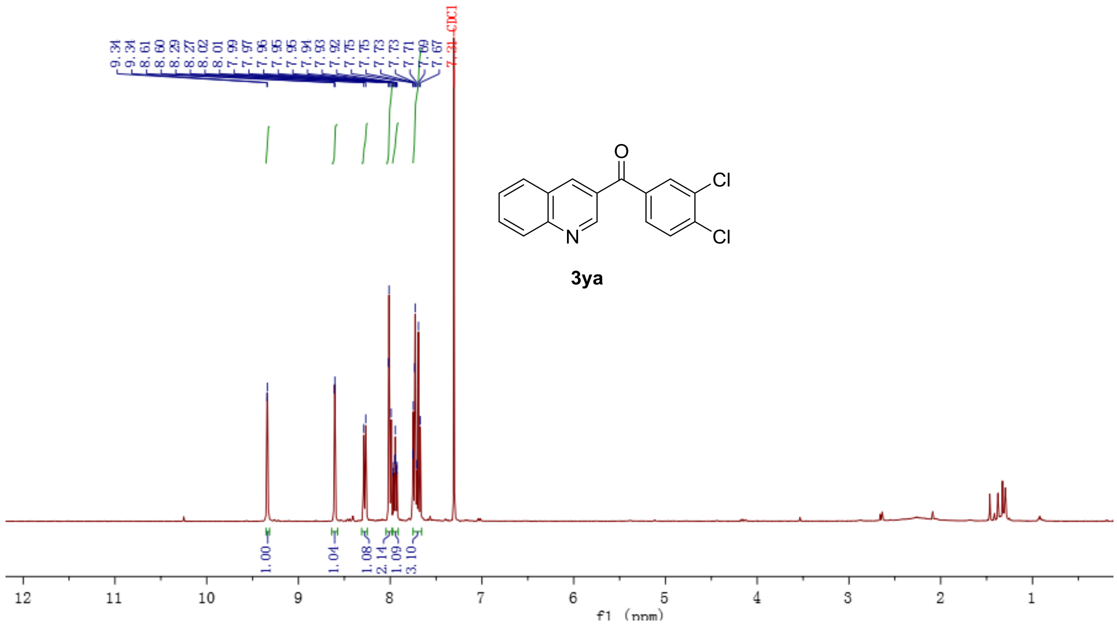
9.34
9.34
8.61
8.60
8.29
8.27
8.02
8.01
7.99
7.97
7.96
7.95
7.95
7.94
7.93
7.92
7.75
7.75
7.73
7.73
7.71
7.69
7.67
7.31 CDCl
O
Cl
N
Cl
3ya
1.00
1.04
1.08
2.14
1.09
3.10
12
11
10
9
8
7
6
5
4
3
2
1
f1 (ppm)

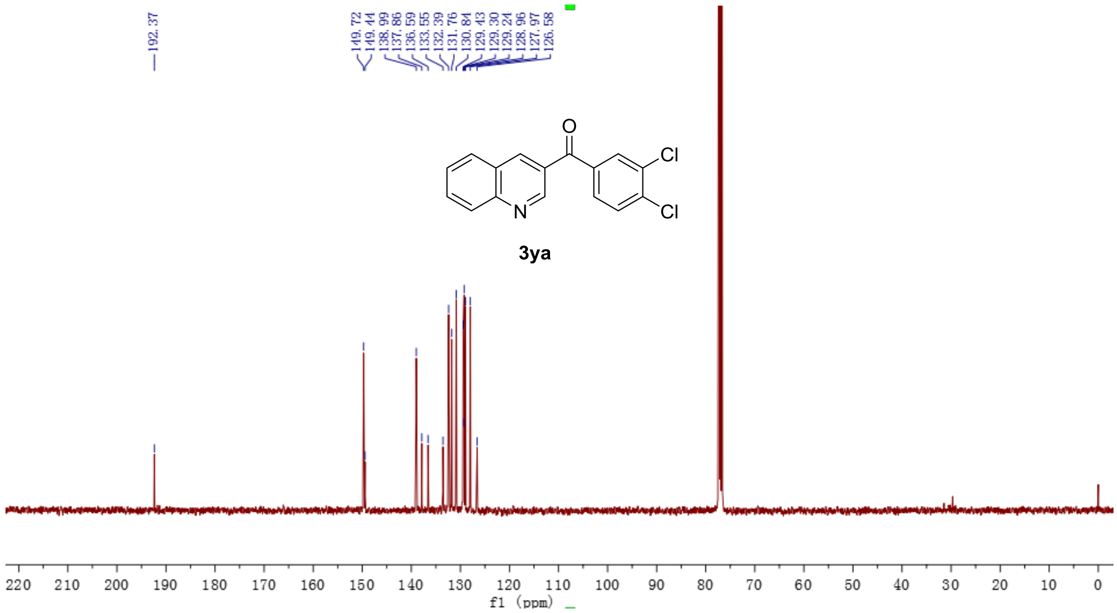
192.37
149.72
149.44
138.99
137.86
136.59
133.55
132.39
131.76
130.84
129.43
129.30
129.24
128.96
127.97
126.58
O
Cl
N
Cl
3ya
220
210
200
190
180
170
160
150
140
130
120
110
100
90
80
70
60
50
40
30
20
10
0
f1 (ppm)

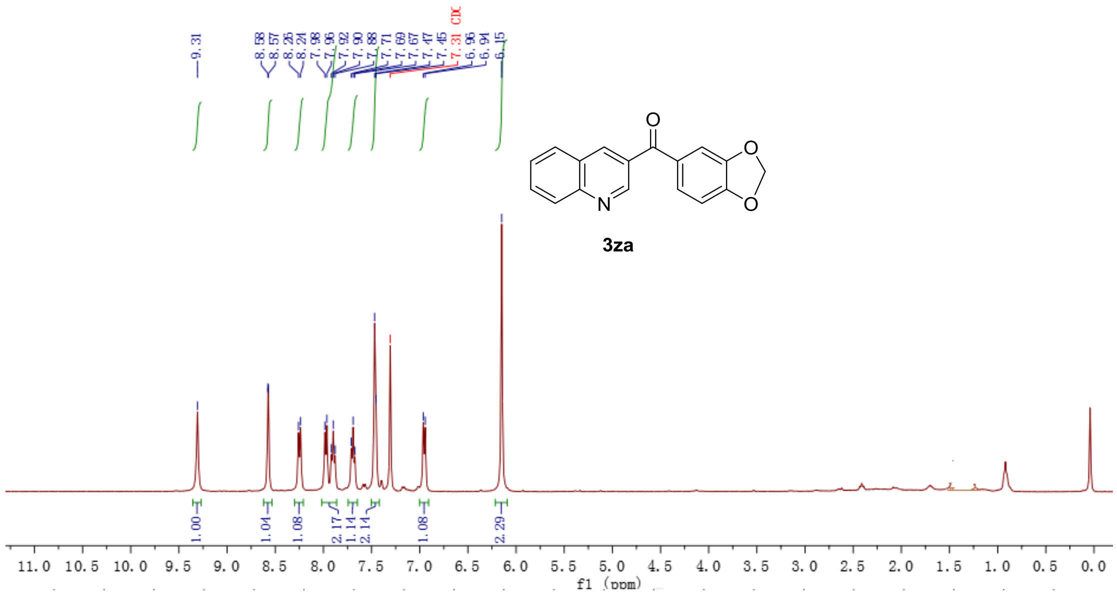
3za
f1 (ppm)

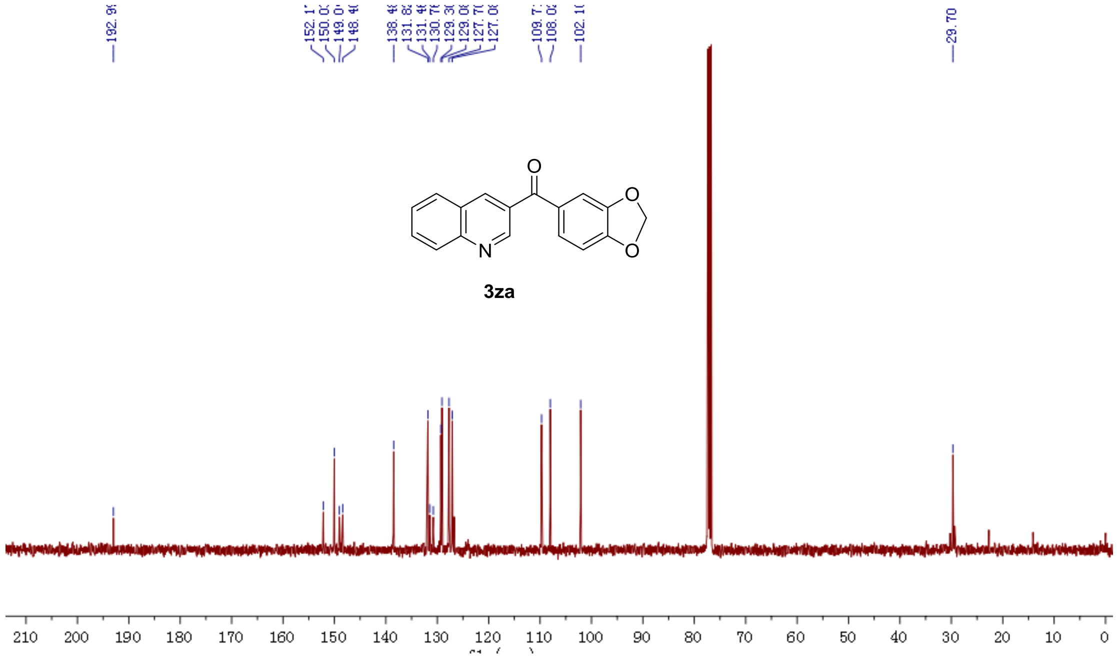
3za
192.9
29.70

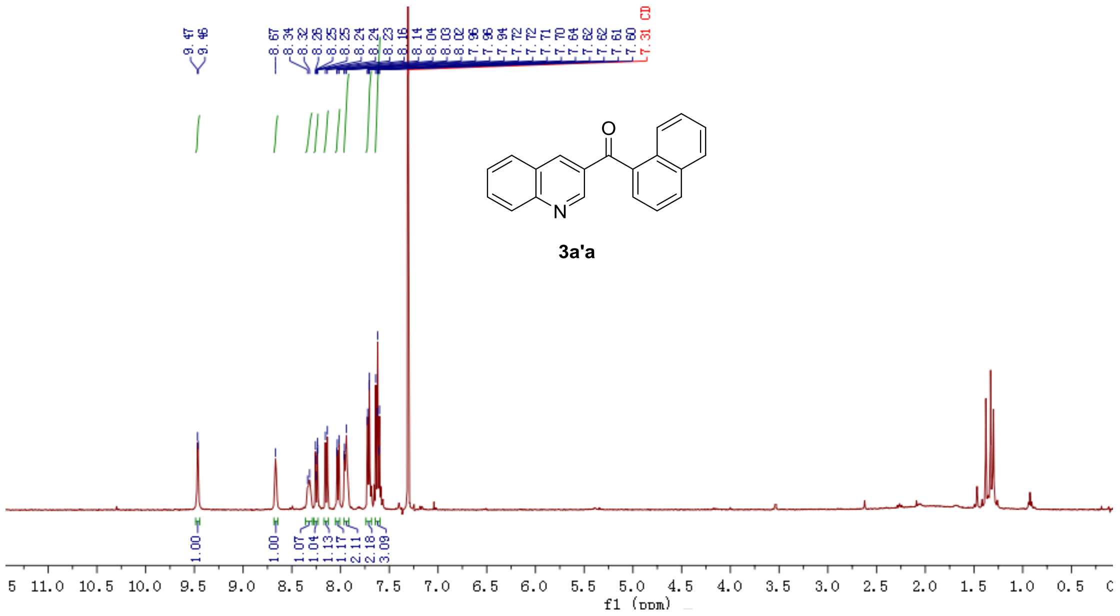
9.47
9.46
8.67
8.34
8.32
8.26
8.25
8.25
8.24
8.24
8.23
8.16
8.14
8.04
8.03
8.02
7.96
7.96
7.94
7.72
7.72
7.71
7.70
7.64
7.62
7.62
7.61
7.60
7.31 CD
O
N
3a'a
1.00
1.00
1.07
1.04
1.13
1.17
2.11
2.18
3.09
f1 (ppm)

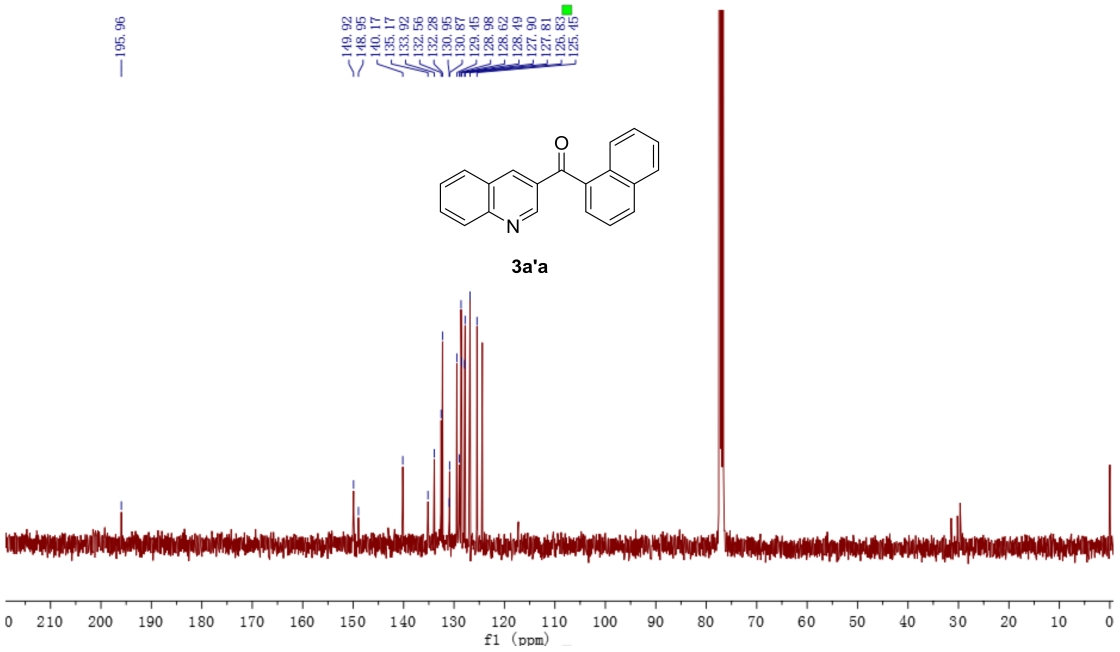
195.96
149.92
148.95
140.17
135.17
133.92
132.56
132.28
130.95
130.87
129.45
128.98
128.62
128.49
127.90
127.81
126.83
125.45
O
N
3a'a
f1 (ppm)

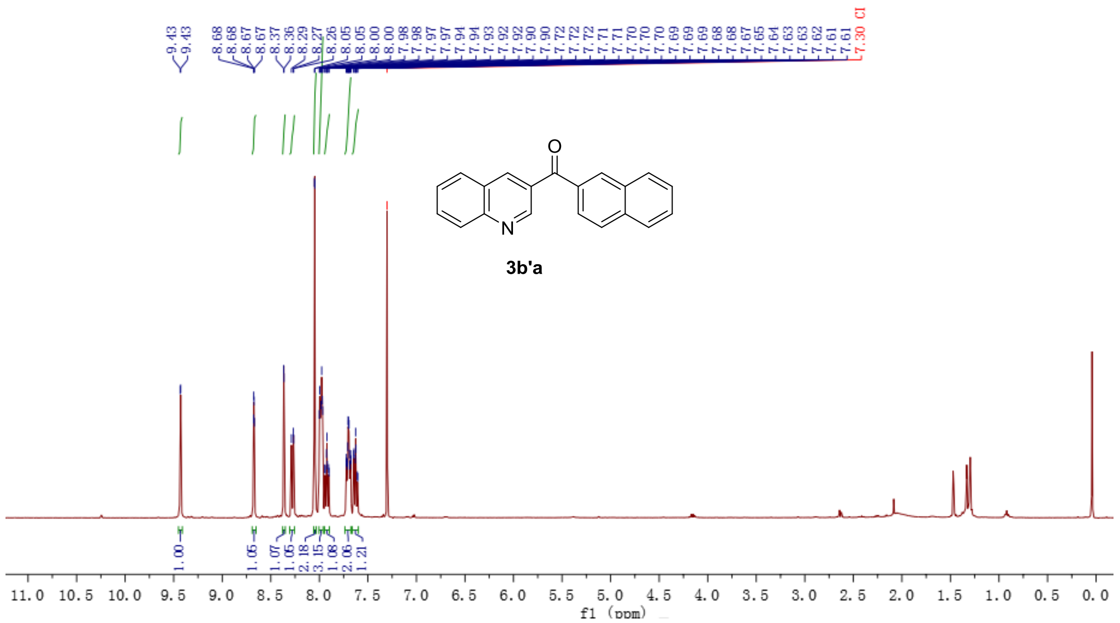
3b'a
f1 (ppm)

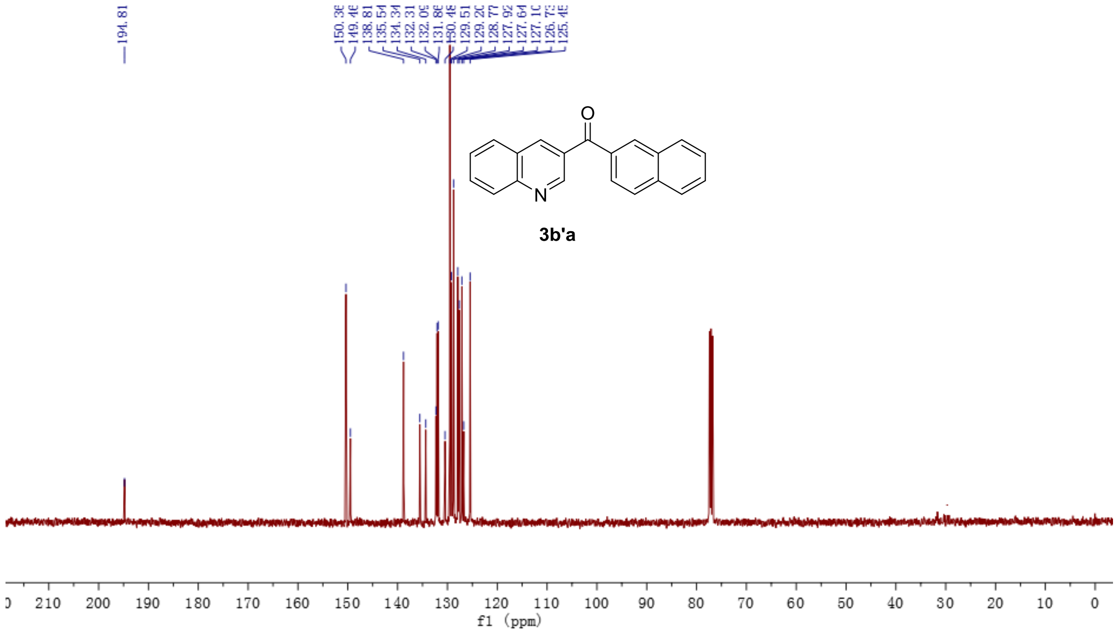
3b'a
f1 (ppm)

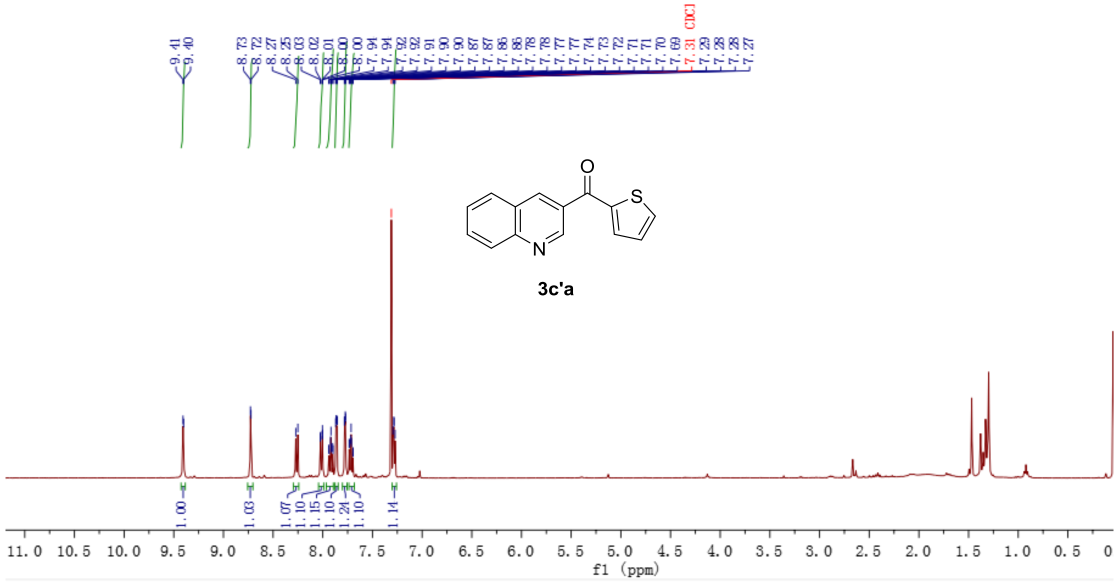

9.41
9.40
8.73
8.72
8.27
8.25
8.03
8.02
8.01
8.00
8.00
7.94
7.94
7.92
7.92
7.91
7.90
7.90
7.87
7.87
7.86
7.86
7.78
7.78
7.77
7.77
7.74
7.73
7.72
7.71
7.71
7.70
7.69
7.31 CDCl
7.29
7.28
7.28
7.27
O
S
N
3c'a
1.00
1.03
1.07
1.10
1.15
1.10
1.24
1.10
1.14
11.0 10.5 10.0 9.5 9.0 8.5 8.0 7.5 7.0 6.5 6.0 5.5 5.0 4.5 4.0 3.5 3.0 2.5 2.0 1.5 1.0 0.5 0
f1 (ppm)

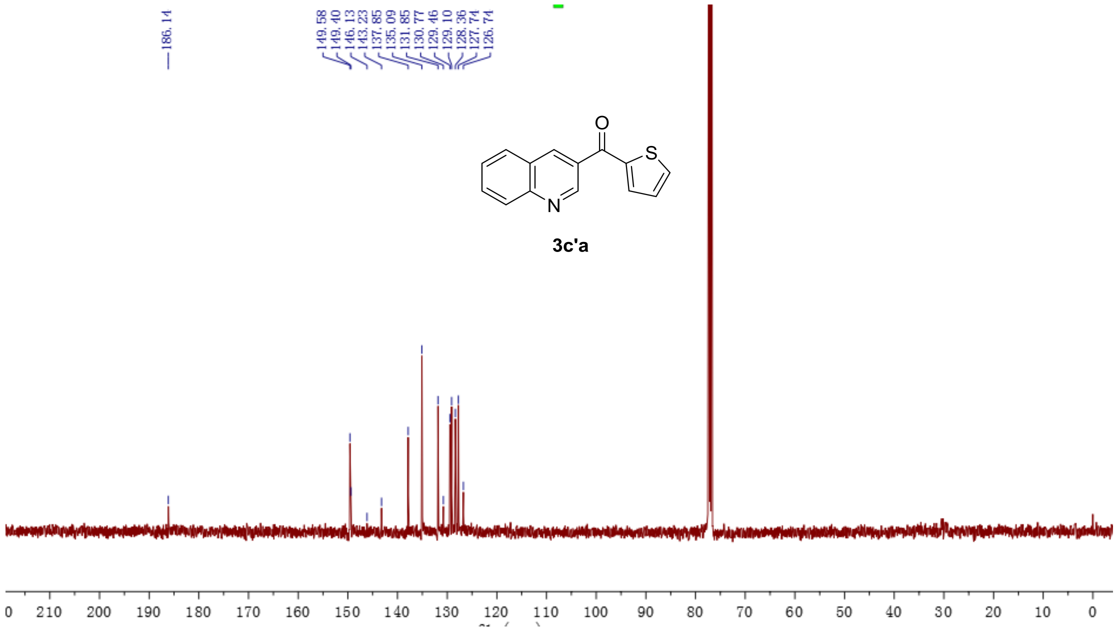

186.14
149.58
149.40
146.13
143.23
137.85
135.09
131.85
130.77
129.46
129.10
128.36
127.74
126.74
O
S
N
3c'a
0 210 200 190 180 170 160 150 140 130 120 110 100 90 80 70 60 50 40 30 20 10 0

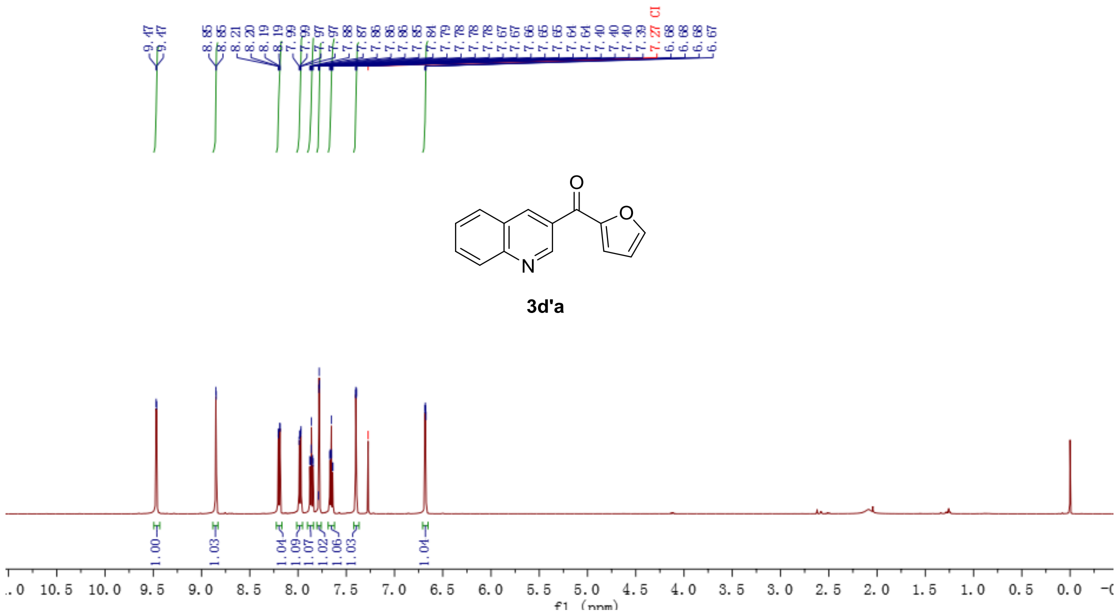

3d'a

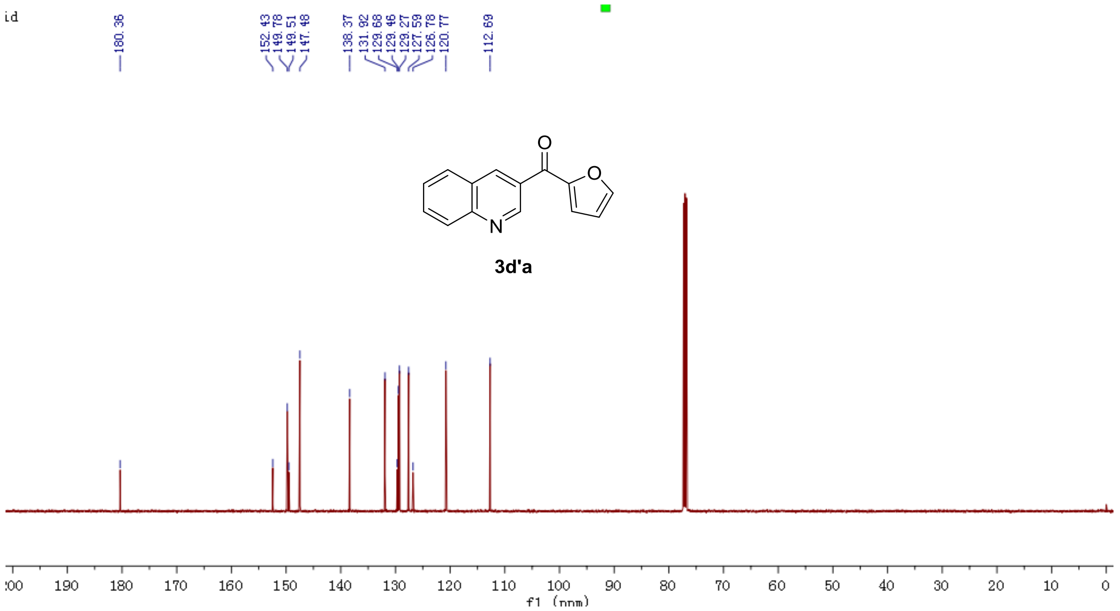

3d'a

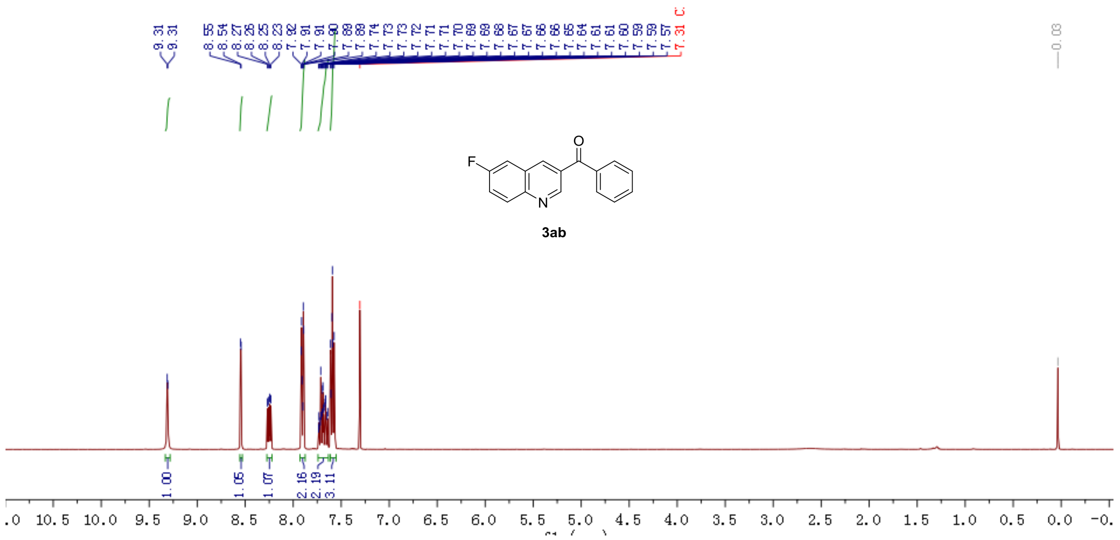

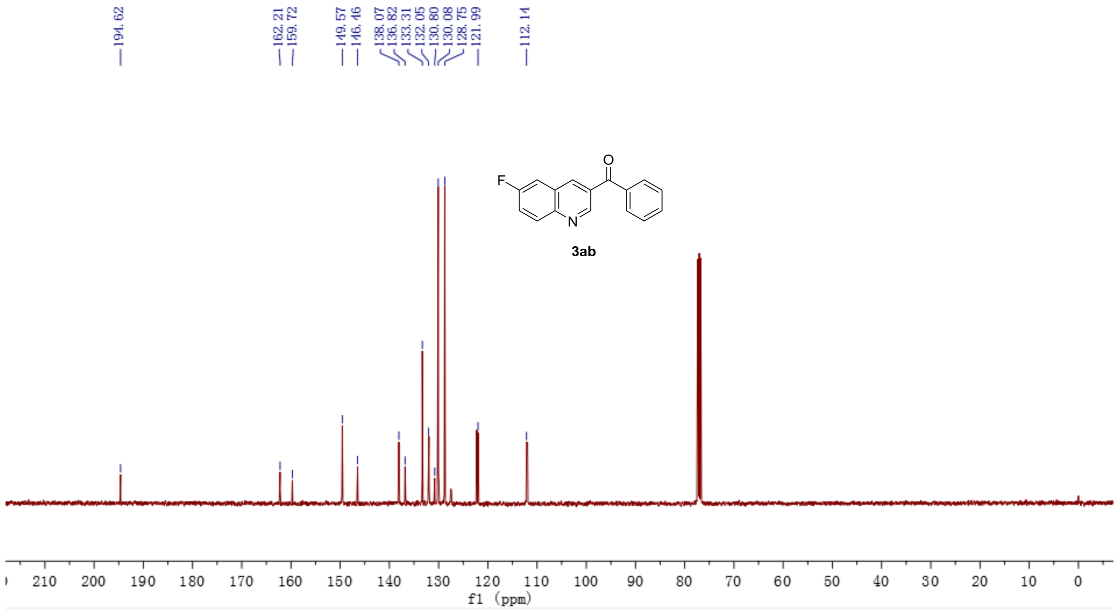

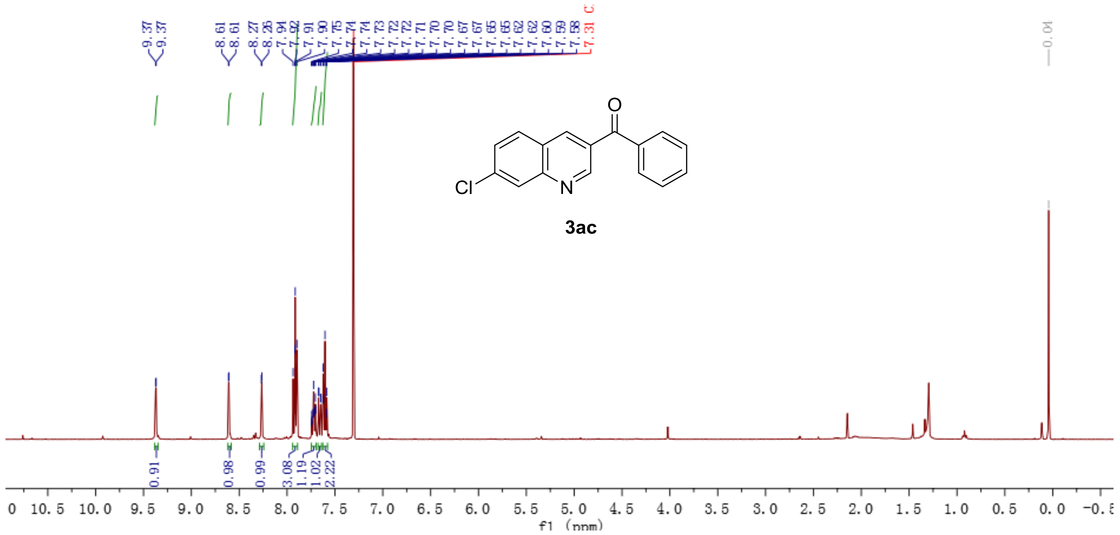
9.37
9.37
8.61
8.61
8.27
8.26
7.94
7.92
7.91
7.90
7.75
7.74
7.74
7.73
7.72
7.72
7.71
7.70
7.70
7.67
7.67
7.65
7.65
7.62
7.62
7.60
7.59
7.58
7.31
0.04
O
Cl
N
3ac
0.91
0.98
0.99
3.08
1.19
1.02
2.22
f1 (ppm)

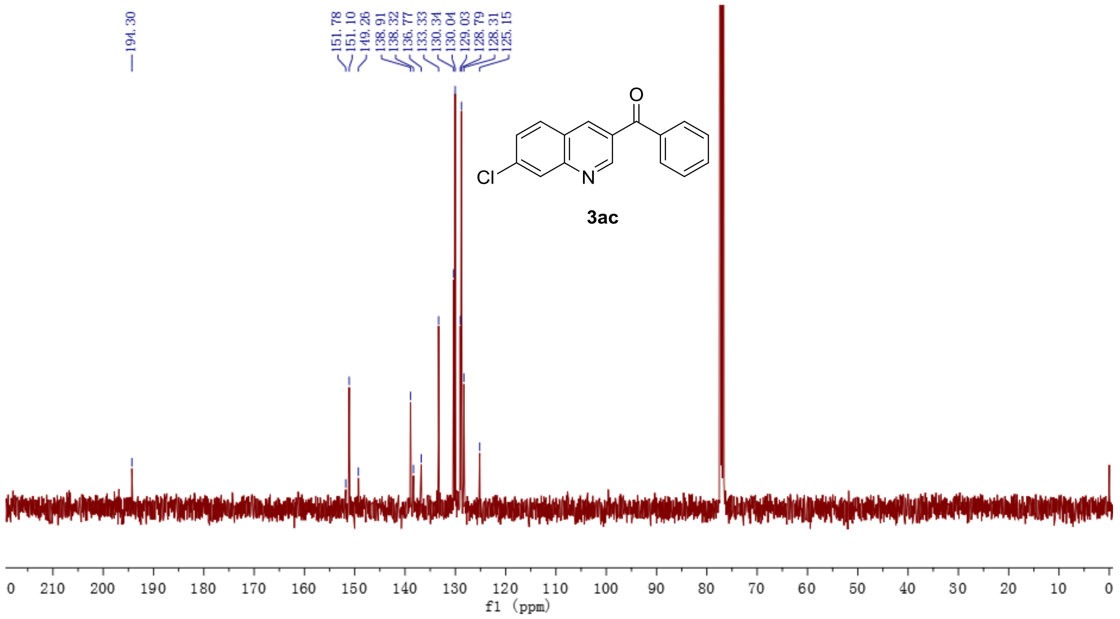
194.30
151.78
151.10
149.26
138.91
138.32
136.77
133.33
130.34
130.04
129.03
128.79
128.31
125.15
O
Cl
N
3ac
f1 (ppm)

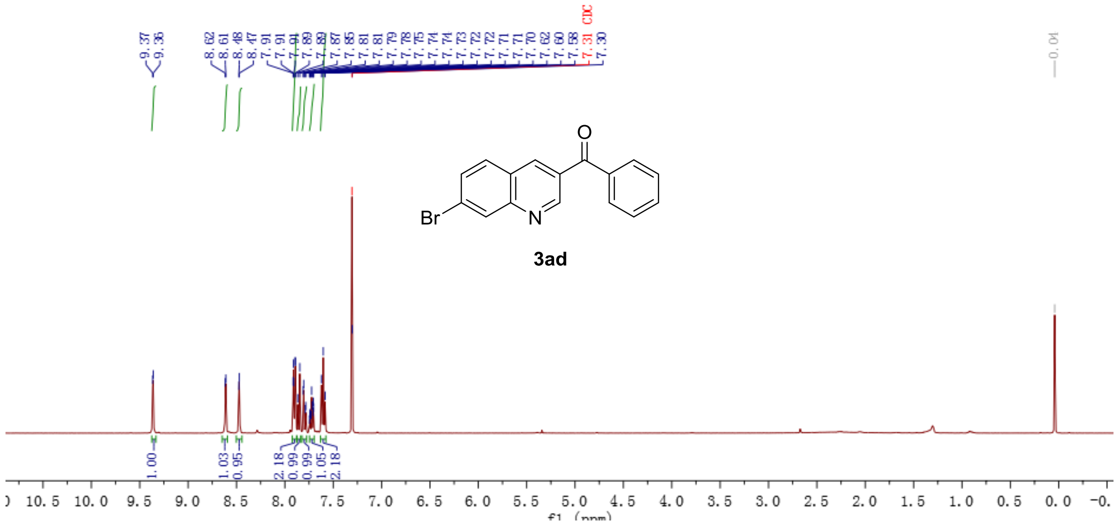
9.37
9.36
8.62
8.61
8.48
8.47
7.31 CDC
7.30
0.04
Br
N
O
3ad
1.00
1.03
0.95
2.18
0.99
0.99
0.99
1.05
2.18
f1 (ppm)

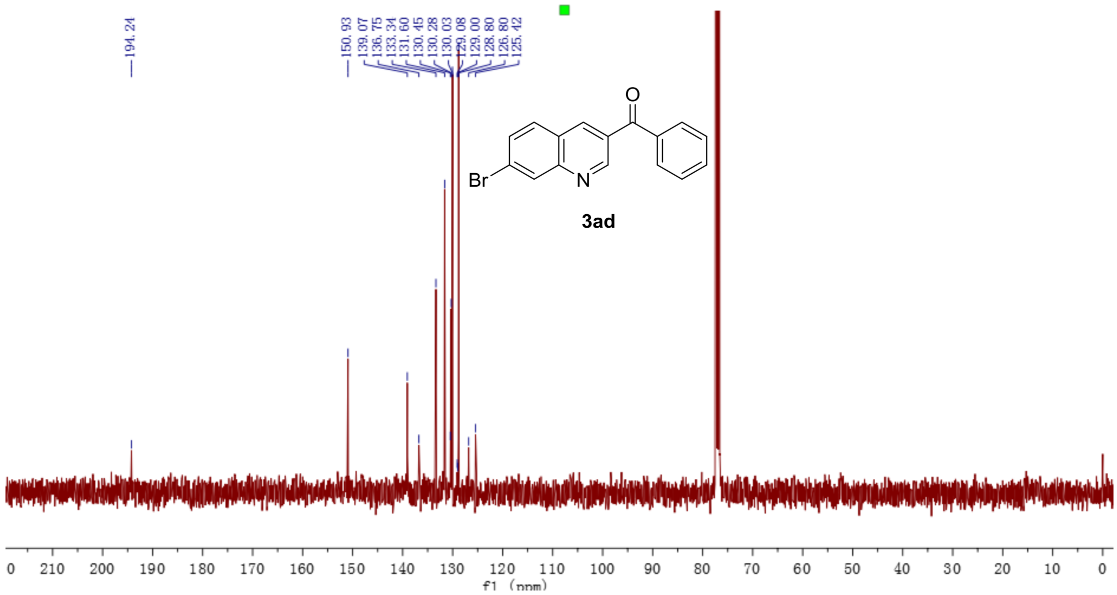
194.24
150.93
139.07
136.75
133.34
131.60
130.45
130.28
130.03
129.08
129.00
128.80
126.80
125.42
Br
N
O
3ad
f1 (ppm)

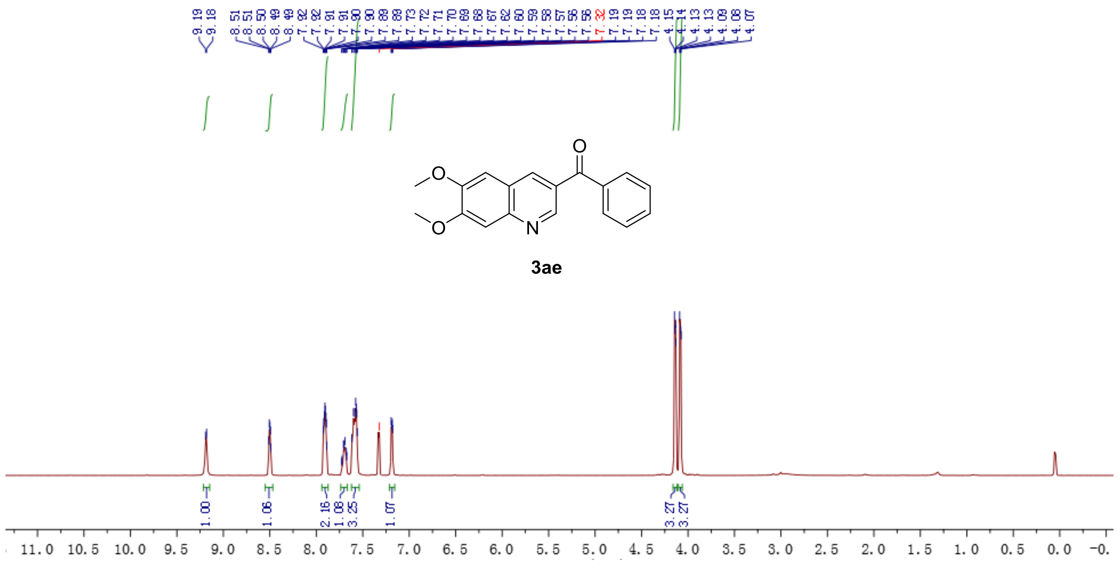
3ae
9.19
9.18
1.00
1.06
2.16
1.08
3.25
1.07
3.27
3.27

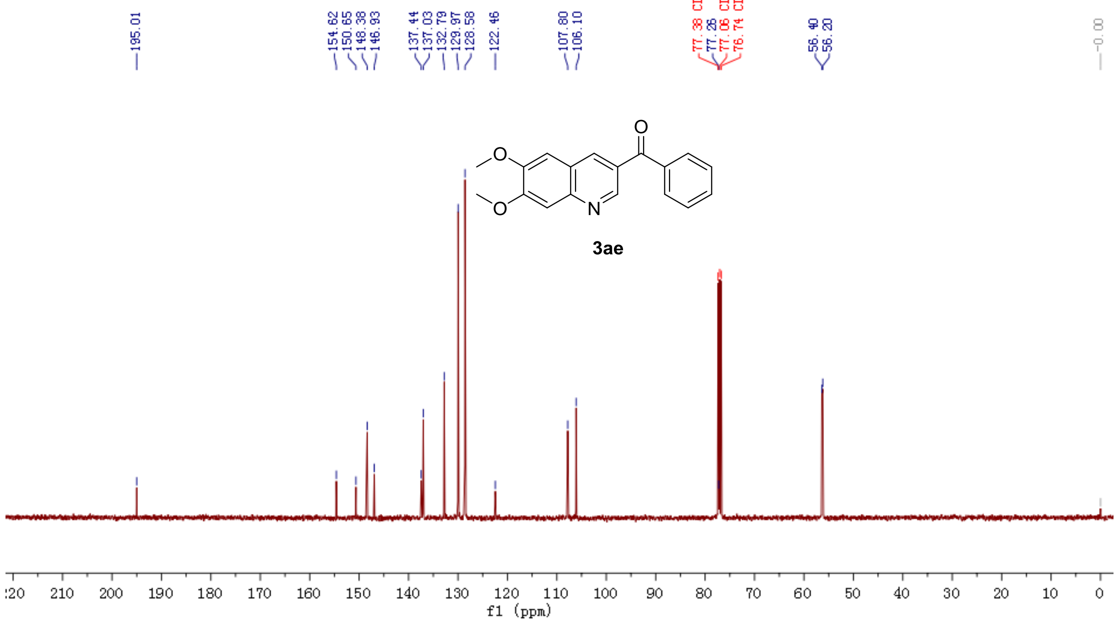
195.01
154.62
150.65
148.38
146.93
137.44
137.03
132.79
129.97
128.58
122.46
107.80
106.10
56.40
56.20
3ae
f1 (ppm)

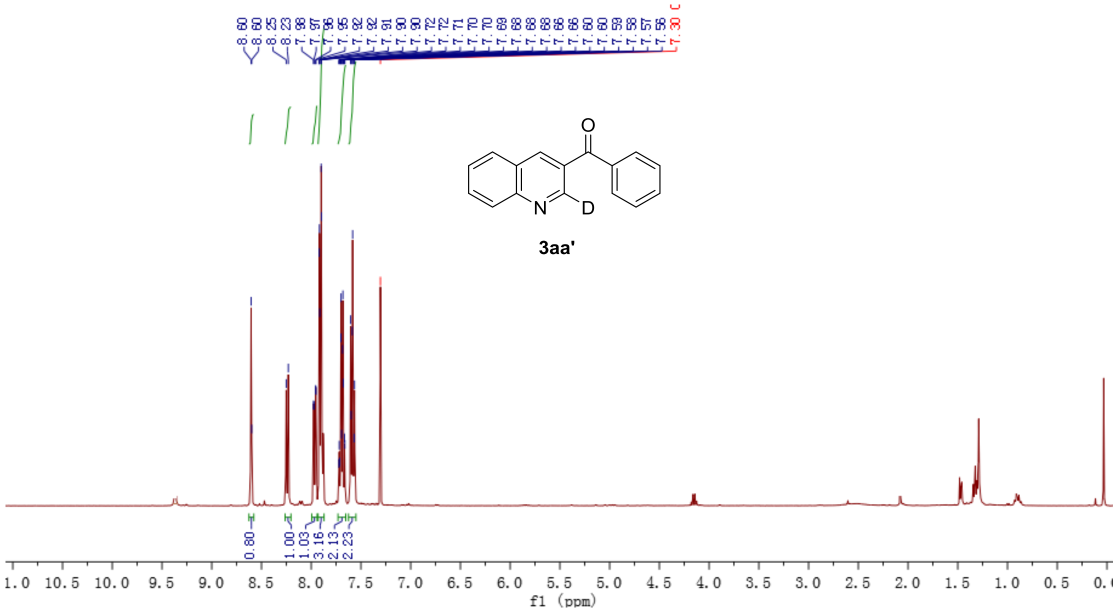

8.60
8.60
8.25
8.23
7.98
7.97
7.96
7.95
7.92
7.92
7.91
7.90
7.90
7.72
7.72
7.71
7.70
7.70
7.69
7.68
7.68
7.68
7.66
7.66
7.60
7.60
7.59
7.58
7.57
7.56
7.30
3aa'
0.80
1.00
1.03
3.16
2.13
2.23
f1 (ppm)

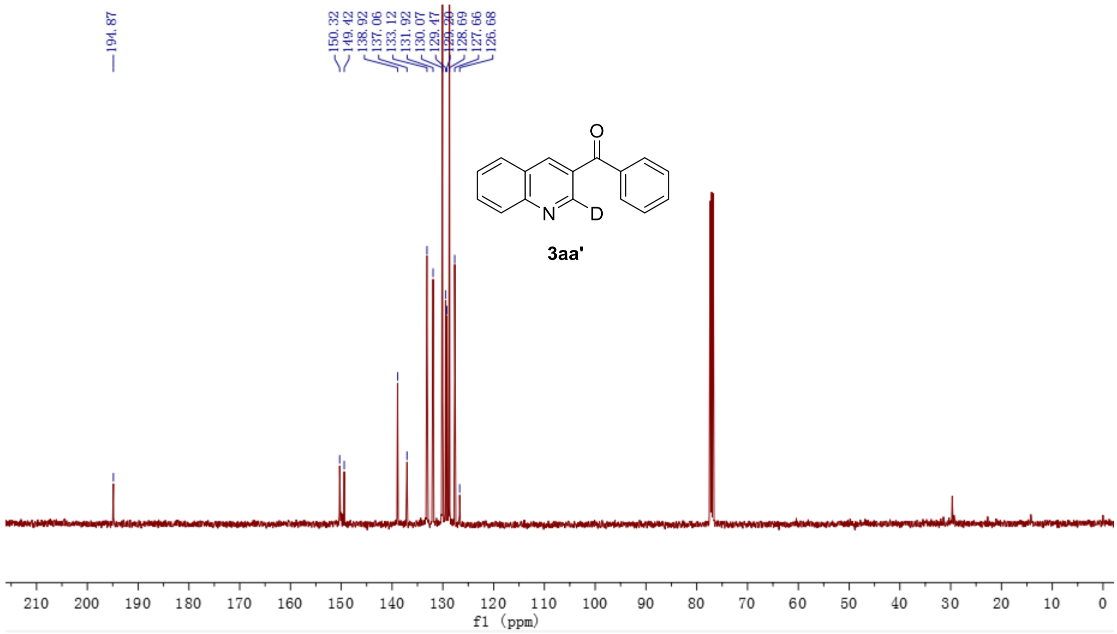

194.87
150.32
149.42
138.92
137.06
133.12
131.92
130.07
129.47
128.69
127.66
126.68
3aa'
f1 (ppm)

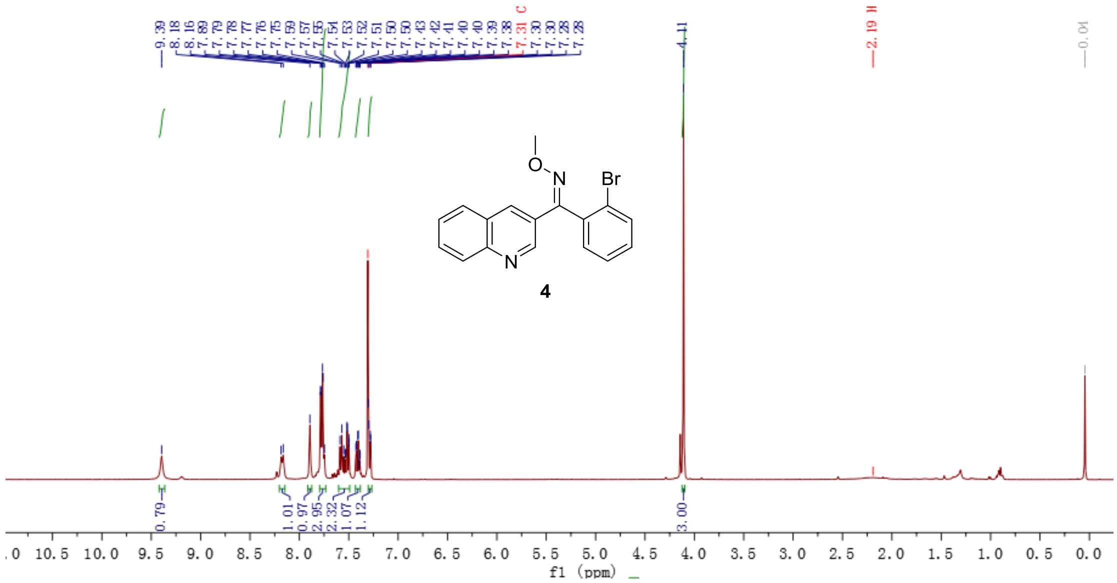
O
N
Br
N
4
f1 (ppm)

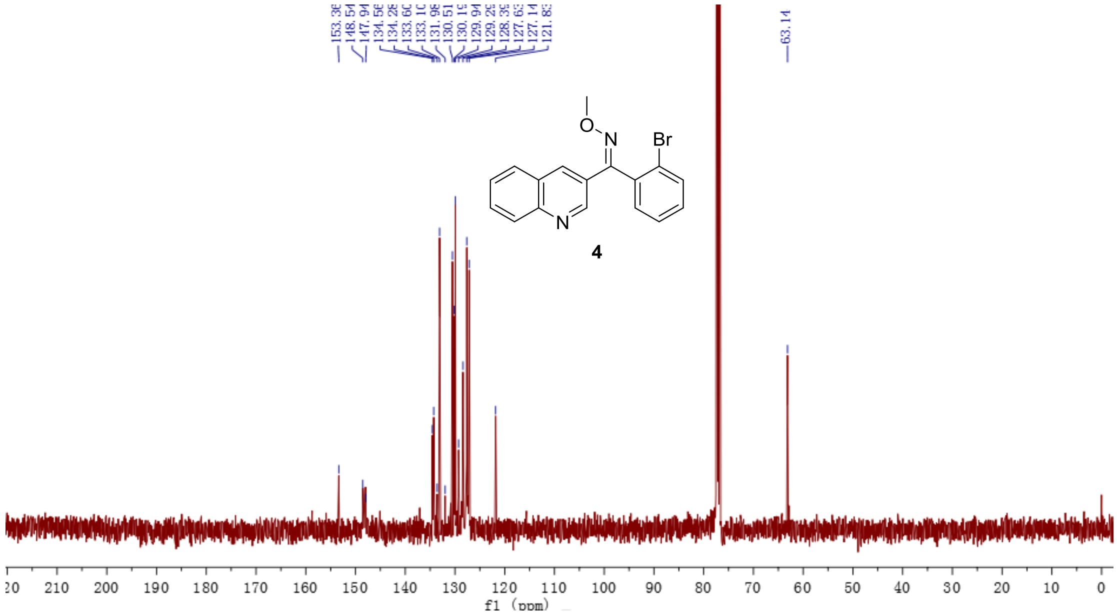
63.14
O
N
Br
N
4
f1 (ppm)
